# Exploring Artificial Intelligence Models for the Detection and Prediction of Sarcopenia: A Scoping Review

**DOI:** 10.3390/jcm15218320

**Published:** 2026-10-26

**Authors:** Luis Armando Solano-Sandi, Eduardo Gallegos, Cristian Barros-Osorio, Jennifer Campos-Aguayo, Isabel Barrientos-Calvo, Fernanda García, Juan José Valenzuela Fuenzalida, Juan Sanchis-Gimeno, Jose E. León-Rojas, Walter Sepúlveda-Loyola

**Affiliations:** 1 Hospital Nacional de Geriatría y Gerontología, Caja Costarricense del Seguro Social, San José 10103, Costa Rica; 2 Grupo de Estudios del Envejecimiento (GEE), Santiago 7500975, Chilefernanda.garcia.g@usach.cl (F.G.);; 3 Complejo Asistencial Dr. Sótero del Río, Santiago 8207257, Chile; 4 Centro de Rehabilitación La Florida, Caja de Previsión de la Defensa Nacional (CAPREDENA), Santiago 8310473, Chile; 5 Programa de Especialidad de Geriatría, Universidad de Santiago de Chile, Santiago 7500975, Chile; 6 Programa de Doctorado en Bienestar Humano, Salud y Sociedad, Facultad de Salud y Ciencias Sociales, Universidad de Las Americas (UDLA), Santiago 7500975, Chile; 7 Unidad de Investigación en Salud, Hospital Nacional de Geriatría y Gerontología, Caja Costarricense del Seguro Social, San José 10103, Costa Rica; ibarrien@ccss.sa.cr; 8 Faculty of Medicine, Andrés Bello University, Santiago 8370146, Chile; 9 Department of Chemical and Biological Sciences, Faculty of Health Sciences, Bernardo O’Higgins University, Santiago 8370933, Chile; 10 GIAVAL Research Group, Department of Anatomy and Human Embryology, Faculty of Medicine, University of Valencia, 46001 Valencia, Spain; juan.sanchis@uv.es; 11 Grupo de Investigación Bienestar, Salud y Sociedad, Escuela de Psicología y Educación, Universidad de Las Américas, Quito 170124, Ecuador

**Keywords:** machine learning, deep learning, screening, risk assessment, skeletal muscle, geriatric assessment, TRIPOD+A

## Abstract

**Background/Objectives:** Sarcopenia is a progressive skeletal muscle disorder associated with adverse health outcomes, including disability, hospitalization, and mortality. Despite the availability of consensus-based diagnostic criteria, its identification in clinical practice remains limited due to reliance on specialized assessments and resource-intensive measurements. Artificial intelligence (AI) and machine learning (ML) approaches have emerged as promising tools to enhance sarcopenia detection; however, evidence remains methodologically heterogeneous and has not been comprehensively synthesized. The objective was to systematically map the existing literature on AI/ML models developed to detect or predict sarcopenia, describing their methodological characteristics, input features, model performance, validation strategies, and adherence to reporting standards. **Methods:** A scoping review was conducted following PRISMA-ScR guidelines. Searches were performed in five databases (MEDLINE, Scopus, SciELO, Web of Science, and LILACS) from inception to 28 February 2026. Studies were included if they developed and/or validated AI/ML models for binary classification of sarcopenia using recognized consensus definitions. Two reviewers independently screened studies, extracted data, and assessed adherence to TRIPOD+AI reporting recommendations and appraised methodological quality using the NHLBI Quality Assessment Tool. Findings were synthesized descriptively. The review protocol was registered in the Open Science Framework. **Results:** Of 2910 records, 28 studies (n = 34,498 participants in the primary analytical cohorts, with an additional 2800 participants in independent external validation cohorts) were included, predominantly from Asia (China n = 14, South Korea n = 7). Random Forest was the most frequent algorithm (n = 6), followed by voting classifiers, MLP, and XGBoost (n = 4 each). Clinical tabular data, including anthropometric and laboratory variables, were the most common input features. Model performance was generally high, with AUC values ranging from 0.710 to 0.975 (mean ≈ 0.869), although heterogeneity across populations and validation strategies was substantial. External validation was performed in only 7 of the 28 studies (25%). No study fully adhered to TRIPOD+AI recommendations; mean compliance was moderate, with critical gaps in code sharing, patient/public involvement, external validation, and subgroup performance reporting. **Conclusions:** AI/ML models, particularly ensemble methods using accessible clinical variables, showed high discrimination in model development settings, with performance estimates that derive predominantly from internal validation and are not comparable across studies. However, important methodological limitations, including lack of external validation, limited generalizability, and suboptimal adherence to reporting standards, were observed. Future research should prioritize external validation in diverse populations, transparent reporting following TRIPOD+AI, and open sharing of code and data to facilitate clinical translation.

## 1. Introduction

Sarcopenia is a progressive and generalized skeletal muscle disorder characterized by reduced muscle strength, low muscle quantity or quality, and impaired physical performance, and is associated with increased risk of falls, disability, hospitalization, and mortality [1]. With the rapid aging of the global population, sarcopenia has emerged as a major public health concern, particularly in low- and middle-income countries where demographic transitions are occurring at an unprecedented pace [2]. Early detection is therefore critical to enable timely preventive and therapeutic interventions.

In recent years, sarcopenia has been consistently defined by major international consensus groups as a condition characterized by the combination of reduced muscle strength, decreased muscle mass or quality, and impaired physical performance. This conceptual framework has been adopted by key organizations, including the European Working Group on Sarcopenia in Older People (EWGSOP2) [1], the Asian Working Group for Sarcopenia (AWGS2) [3], and the Foundation for the National Institutes of Health (FNIH) Sarcopenia Project [4], which have operationalized these components into diagnostic criteria. More recently, the Global Leadership Initiative on Sarcopenia (GLIS, 2024) has sought to harmonize these approaches by providing a globally applicable definition and unified diagnostic framework [5]. Despite these advances, the application of these criteria in routine clinical practice remains limited. Barriers include restricted access to imaging technologies, variability in cut-off values across populations, operator dependency, and the need for specialized equipment [6]. Consequently, sarcopenia remains underdiagnosed. In an international survey of 255 clinicians from 55 countries, only about half reported assessing muscle mass or muscle strength in routine practice in adults aged 60 years and older, and the tools and cut-off values applied varied widely [7].

In this context, artificial intelligence (AI) and machine learning (ML) approaches have been increasingly explored to enhance sarcopenia screening, classification, and prediction [8]. These models offer the potential to identify non-linear relationships, integrate multimodal inputs, and improve diagnostic accuracy beyond traditional rule-based algorithms [8]. However, the rapid growth of AI applications in this field has produced a heterogeneous body of literature, with variability in data sources, model architectures, validation strategies, and reported performance metrics.

Because these terms are frequently used interchangeably in the AI literature, we adopt the following operational distinctions throughout this review. Screening refers to the use of simple, low-burden instruments to identify individuals with a high probability of sarcopenia who should undergo formal assessment. Detection, or case finding, refers to the classification of prevalent sarcopenia at the time of assessment, using a consensus definition as the reference standard. Prediction refers to the estimation of the probability that sarcopenia will develop at a future time point in individuals who do not meet the criteria at baseline. Diagnosis refers to the confirmatory classification obtained by applying the full consensus criteria of muscle strength, muscle quantity or quality, and physical performance; it constitutes the reference standard against which models are developed and evaluated, rather than a task performed by the models themselves. Similarly, we use artificial intelligence as the umbrella term, machine learning for algorithms that learn patterns from data without explicit rule-based programming, and deep learning for the subset of machine learning based on multilayer neural networks.

Previous work has addressed the application of AI to sarcopenia, primarily through broad narrative overviews of available AI approaches and their potential applications [8,9]. However, these reviews did not systematically evaluate reporting completeness using TRIPOD+AI or jointly examine methodological quality, external validation, class imbalance handling, calibration reporting, and code availability. Given the methodological diversity and evolving nature of this research area, a scoping review is warranted to systematically explore and categorize existing evidence, identify research gaps, and inform future development and translational implementation of AI-based detection and prediction tools. Accordingly, the objective of this scoping review is to systematically map the existing literature on AI and ML models developed for the detection or prediction of sarcopenia, describing their methodological characteristics, input features, model performance, validation strategies, methodological quality, and adherence to TRIPOD+AI reporting recommendations. Beyond mapping the available models and their classification performance, this review provides a comprehensive appraisal of key methodological issues, including external validation, class imbalance management, model transparency, and reproducibility. By integrating these dimensions, this review identifies critical methodological and reporting gaps that may influence model generalizability and clinical implementation, thereby providing guidance for the development and validation of more robust AI-based approaches for sarcopenia detection.

## 2. Materials and Methods

We performed this scoping review in accordance with the JBI methodology for scoping reviews [10]. It is reported and summarized using the Preferred Reporting Items for Systematic Reviews and Meta-Analyses (PRISMA) extension for scoping reviews (PRISMA-ScR, 2022) [11] The protocol for this scoping review was registered on OSF on 18 February 2026. (https://osf.io/ptzcb/overview accessed on 1 March 2026). The protocol was registered before the searches, study selection, data extraction, and synthesis. No eligibility criterion or outcome was modified after the search results were known.

The review question was structured according to the Population, Concept, and Context (PCC) framework recommended for scoping reviews. Population: adults in whom sarcopenia was ascertained according to a recognized international consensus definition. Concept: artificial intelligence or machine learning models producing a binary classification of sarcopenia (present versus absent). Context: any clinical or community setting, in any country.

The questions of the scoping review were:

1. What AI/ML algorithms have been used for binary classification of sarcopenia, and how have these evolved over time?
2. What are the most frequently used input variables/features (e.g., DXA/CT/MRI/ultrasound, BIA, strength tests, gait/performance, labs, EHR variables)?
3. What are the reported sensitivity and specificity (and other metrics), and how are they estimated (internal/external validation)?
4. What is the level of adherence of the included studies to the TRIPOD+AI reporting recommendations for the development and/or evaluation of artificial intelligence-based prediction models?

Two deviations from the registered protocol are reported here in the interest of transparency. First, the formal appraisal of methodological quality using the NHLBI Quality Assessment Tool was introduced during peer review and was not pre-specified in the protocol. Second, the stratified descriptive comparison of performance by diagnostic consensus criterion (Supplementary Table S2) was likewise added post hoc in response to peer review. No changes were made to the eligibility criteria, the search strategy, or the primary review questions after registration.

### 2.1. Search Strategy and Inclusion Criteria

The scoping review search was conducted across five databases from inception to 28 February 2026: MEDLINE (via PubMed, National Library of Medicine), Scopus (Elsevier), Web of Science Core Collection (Clarivate), SciELO, and LILACS (via the Virtual Health Library, BIREME/PAHO/WHO). All databases were searched on the same date. The search strategy, including all identified keywords, MeSH (Medical Subject Headings) and DeCS (*Descriptores en Ciencias de la Salud*) terms in English, Portuguese, and Spanish, adapted to the syntax requirements of each interface, is provided in Supplementary Table S1. The reference list of all included sources of evidence was screened for additional studies.

Eligibility was restricted to studies published in English, Spanish, or Portuguese. This restriction reflects the working languages of the review team and allowed full-text assessment without translation. It was pre-specified in the protocol, and its implications are addressed in the Limitations section.

This scoping review considered development and/or validation studies of prediction models aimed at the binary classification of sarcopenia (present versus absent). Models based on regression methods, such as penalized or multivariable logistic regression, were eligible alongside classical machine learning and deep learning approaches. This decision follows the scope of the TRIPOD+AI statement, which explicitly covers prediction models developed with either regression or machine learning methods [12], and reflects the fact that regression-based models are routinely used as the reference comparator against which machine learning performance is benchmarked in this literature. Excluding them would have produced an incomplete map of the field and would have precluded any appraisal of whether the additional complexity of machine learning translates into better classification performance.

Studies that met the following criteria were included:

(1) Population: Adults assessed for sarcopenia for whom it was explicitly stated that sarcopenia was diagnosed according to a recognized consensus or guideline (e.g., EWGSOP2, AWGS, FNIH, IWGS).
(2) Concept: AI/ML models that output binary sarcopenia classification (present/absent).
(3) Context: Any setting (community, hospital, outpatient), any country.

Studies were excluded if sarcopenia was not defined according to an established consensus; if the AI model predicted continuous outcomes (e.g., muscle mass values) without a binary classification aligned with consensus criteria; if the study did not involve an AI-based model; or if the study was a review, editorial, commentary, or animal study. Screening tools not embedded within an AI or machine learning framework were also excluded.

### 2.2. Study Selection

All records were imported into Rayyan (web-based version, Rayyan Systems Inc., Cambridge, MA, USA) for reference management and duplicate removal. Screening was conducted in two stages: (1) title and abstract screening and (2) full-text eligibility assessment. Both stages were performed independently by two reviewers (LAS and CBO). Discrepancies were resolved through discussion, and when necessary, by consultation with a third reviewer (WSL). The study selection process is presented using a PRISMA-ScR flow diagram. Reasons for exclusion at the full-text stage were documented.

### 2.3. Data Extraction

Data extraction was performed independently by two reviewers using a pilot-tested standardized extraction form developed specifically for this review. The extraction form was iteratively refined after pilot testing on a subset of studies to ensure completeness and alignment with the review objectives.

Extracted data included: (1) study characteristics (author, year, country, design, and setting); (2) participant characteristics (sample size, age, sex distribution, clinical population, and sarcopenia prevalence); (3) sarcopenia reference standard (consensus used and diagnostic components); (4) AI/ML model characteristics (algorithm type, feature selection methods, input variables, handling of missing data, class imbalance management, and hyperparameter tuning); (5) validation strategy (internal validation, cross-validation, and external validation); (6) diagnostic performance metrics (sensitivity, specificity, AUC, calibration metrics, and confidence intervals); (7) model output format (probabilities vs. thresholds); (8) reporting transparency (data/code availability); and (9) adherence to TRIPOD+AI reporting items. Disagreements in data extraction were resolved by consensus or, when necessary, by consultation with a third reviewer. For each study, the best-performing model on the overall sample was used, and its AUC, sensitivity, specificity, and F1-score were extracted. This rule maps the performance that the original authors put forward as their main result; its optimistic implications are addressed in the Limitations section. When a metric was reported only stratified by subgroup (e.g., by sex), the first-reported subgroup value was used, applied consistently across all such studies. When a study reported both a single hold-out result and a cross-validated result for the same final model, the cross-validated estimate was used. When a study reported model performance separately for a validation set and an independent test set, the test set estimate was used, as it reflects performance on data not involved in model selection or hyperparameter tuning. When a metric was reported only as a range across data partitions without a single point estimate, or only per diagnostic sub-criterion without a combined value, it was recorded as not reported (NR).

### 2.4. Data Analysis

Data analysis was primarily descriptive in nature, as the aim of this review was to systematically map the methodological characteristics and reporting practices of artificial intelligence- and machine learning-based models for sarcopenia detection or prediction, rather than to estimate pooled measures of diagnostic accuracy. Descriptive analyses were conducted to summarize the frequency of AI/ML algorithms employed, the distribution of input features used across studies, the types of validation strategies applied, the classification performance metrics reported, and the proportion of TRIPOD+AI reporting items fulfilled. The findings are presented using tabular and graphical formats to provide an overview of the existing evidence base and to identify methodological patterns, reporting gaps, and areas requiring further research.

Algorithms were grouped into families for descriptive purposes. Ensemble methods combine multiple base learners to reduce variance or bias, either by aggregating independently trained trees (Random Forest), by sequentially fitting each learner to the residuals of the previous one (gradient boosting, XGBoost, LightGBM, and CatBoost), or by pooling the outputs of heterogeneous classifiers (voting classifiers). Neural networks learn hierarchical non-linear representations through layers of weighted units (multilayer perceptron, wide and deep networks). Linear models estimate an additive combination of predictors on a transformed scale (logistic regression, linear discriminant analysis). Metric-based methods classify observations by their proximity to labeled examples in feature space (k-nearest neighbors). The principal technical characteristics of each family are described above.

### 2.5. Concordance with TRIPOD+AI Reporting Recommendations

We assessed whether each included study adhered to the TRIPOD+AI reporting recommendations for the development and/or evaluation of artificial intelligence-based prediction models. For each study, adherence was evaluated at the item level using a standardized checklist adapted from the official TRIPOD+AI framework [13].

Concordance criteria were operationalized across key reporting domains, including: (1) clear identification of the study as developing and/or evaluating a multivariable prediction model, specification of the target population, and definition of the binary diagnostic outcome (presence versus absence of sarcopenia); (2) transparent description of data sources, participant eligibility criteria, reference standard for sarcopenia diagnosis according to an established consensus, and time frame of data collection; (3) explicit definition of predictors and outcome measures, including how and when they were assessed; (4) comprehensive reporting of analytical methods, including model type, feature handling, hyperparameter tuning, validation strategy (internal and/or external), and performance metrics such as sensitivity, specificity, and discrimination measures; and (5) reporting of model performance with appropriate confidence intervals and, where applicable, subgroup analyses or heterogeneity assessments. Additional TRIPOD+AI domains evaluated included handling of missing data, management of class imbalance, reporting of model output format (e.g., probabilities or classification thresholds), transparency regarding data and code availability, and disclosure of funding and conflicts of interest.

For each study, individual checklist items were categorized as “reported” or “not reported.” Overall adherence was summarized descriptively as the proportion of fulfilled TRIPOD+AI items relative to the total applicable items for development and/or evaluation studies. No minimum threshold was imposed for study inclusion; however, reporting completeness was analyzed to identify common areas of underreporting and methodological limitations within the field.

### 2.6. Methodological Quality Assessment

The methodological quality of the included studies was assessed using the National Heart, Lung, and Blood Institute (NHLBI) Quality Assessment Tool for Observational Cohort and Cross-Sectional Studies. The tool comprises 14 criteria addressing key methodological domains, including clarity of the research question, definition of the study population, participant selection, sample size justification, temporal relationship between exposure and outcome, exposure and outcome measurement, repeated exposure assessment, blinding of outcome assessors, loss to follow-up, and statistical adjustment for potential confounding variables. Each criterion was classified as “Yes” (Y), “No” (N), “Not Reported” (NR), or “Not Applicable” (NA), according to the information provided in the original publication. This instrument was selected because all included studies were, by design, observational cohort or cross-sectional investigations, and because it appraises the study-level features that most directly condition the credibility of any prediction model derived from such data. We note explicitly that the NHLBI tool does not constitute a risk of bias assessment specific to prediction models. Instruments developed for that purpose, namely PROBAST and its recent extension PROBAST+AI [14], evaluate model-specific domains such as predictor handling, adequacy of the modeling strategy, overfitting, and calibration, which the NHLBI tool does not address. Consistent with JBI guidance and with the PRISMA-ScR statement, critical appraisal is an optional component of scoping reviews. In the present review, these model-specific dimensions were addressed through the item-level assessment of adherence to TRIPOD+AI, and the absence of a formal PROBAST+AI assessment is acknowledged as a limitation. The assessment was conducted independently by two reviewers (LAS and CBO), with disagreements resolved through discussion and, where consensus could not be reached, by consultation with a third reviewer (WSL). The assessment was used to characterize the methodological strengths and limitations of the included studies and to support the interpretation of the overall body of evidence.

During the revision of this manuscript, the authors used Claude 5.1 (Anthropic, San Francisco, CA, USA) to assist with English-language editing, refinement of wording, and improvement of the clarity and organization of selected sections of the manuscript and responses to peer review comments. Generative artificial intelligence was not used for study selection, data extraction, data analysis, methodological quality assessment, or generation of the study results. All AI-assisted content was critically reviewed, edited, and verified by the authors against the original sources and study data. The authors take full responsibility for the accuracy and integrity of the final manuscript.

## 3. Results

### 3.1. Study Characteristics

**Figure 1:**
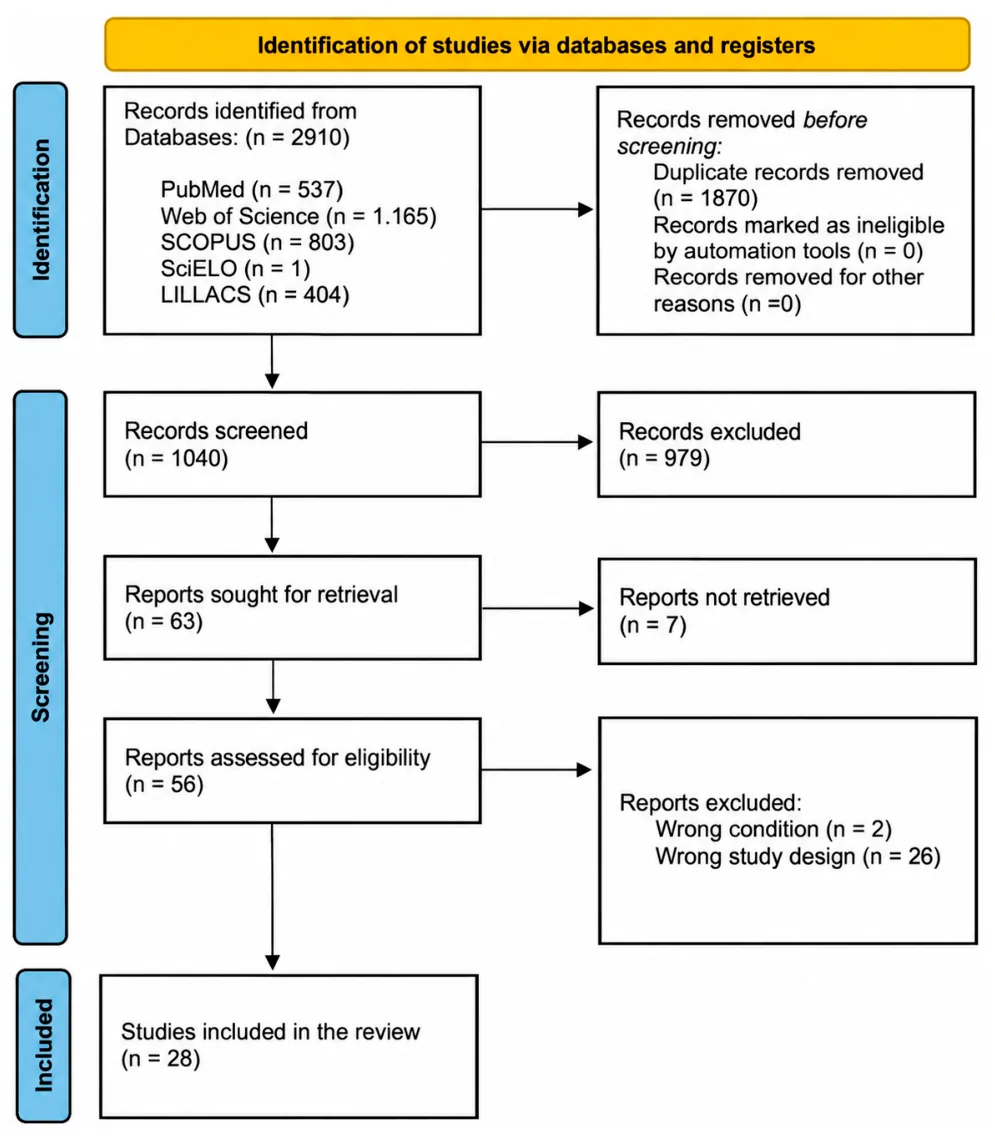
PRISMA Flow search strategy.

Of the 2910 articles identified in our initial search, 28 studies were included in the data analysis, with a total population of 34,498 participants in the primary analytical cohorts and an additional 2800 participants in independent external validation cohorts (Figure 1 presents the details of the search and study selection process). Characteristics of the studies are reported in Table 1. Most included studies involved individuals older than 60 years, with women comprising 54% of participants, and spanned 2021 to 2026. These studies predominantly originated from Asia, with China contributing the highest number (n = 14) [15,16,17,18,19,20,21,22,23,24,25,26,27,28], followed by South Korea (n = 7) [29,30,31,32,33,34,35], the United States (n = 2) [36,37], and single contributions from Denmark (n = 1) [38], India (n = 1) [39], Spain (n = 1) [40], Turkey (n = 1) [41], and Taiwan (n = 1) [42]. Regarding study design, 23 were cross-sectional (including one retrospective cross-sectional study), four were prospective cohort studies, and one was a longitudinal cohort study. The reported prevalence of sarcopenia across the included cohorts ranged widely, from 8.3% to 57.3% [15,16,17,18,19,20,21,22,23,24,25,26,27,28,29,30,31,32,33,34,35,36,37,38,39,40,41,42] (Table 1).

### 3.2. Diagnostic Criteria for Sarcopenia

Diagnostic criteria for sarcopenia varied across the 28 included studies. The majority of the studies used the updated Asian Working Group for Sarcopenia consensus (AWGS2) (n = 21; 75%) [15,16,17,18,19,20,21,22,23,24,25,26,27,28,29,31,32,33,34,35,42], followed by the revised European Working Group on Sarcopenia in Older People criteria (EWGSOP2; n = 6; 21.4%) [30,37,38,39,40,41], and the Foundation for the National Institutes of Health Sarcopenia Project criteria (FNIH; n = 1; 3.6%) [36] (Table 1).

To explore whether performance metrics differed according to diagnostic consensus criterion, AUC, sensitivity, specificity, and F1-score were summarized descriptively by criterion (Supplementary Table S2). When stratified by diagnostic criterion, models based on EWGSOP2 showed a median AUC of 0.924 (range 0.870–0.975; n = 4), a median sensitivity of 0.915 (0.800–1.000; n = 5), a median specificity of 0.922 (0.829–0.987; n = 4), and a median F1-score of 0.833 (0.748–0.921; n = 5). Models based on AWGS2, the most frequently used criterion, showed a median AUC of 0.871 (0.710–0.970; n = 18), a median sensitivity of 0.775 (0.441–1.000; n = 17), a median specificity of 0.882 (0.670–1.000; n = 16), and a median F1-score of 0.778 (0.392–1.000; n = 14). Only one study applied the FNIH criterion [36], reporting an AUC of 0.763 with no other performance metrics, precluding any subgroup summary. Given the small and uneven number of studies across diagnostic criteria, these data are presented as descriptive comparisons only.

### 3.3. Machine Learning Algorithms Used for the Detection of Sarcopenia

**Figure 2:**
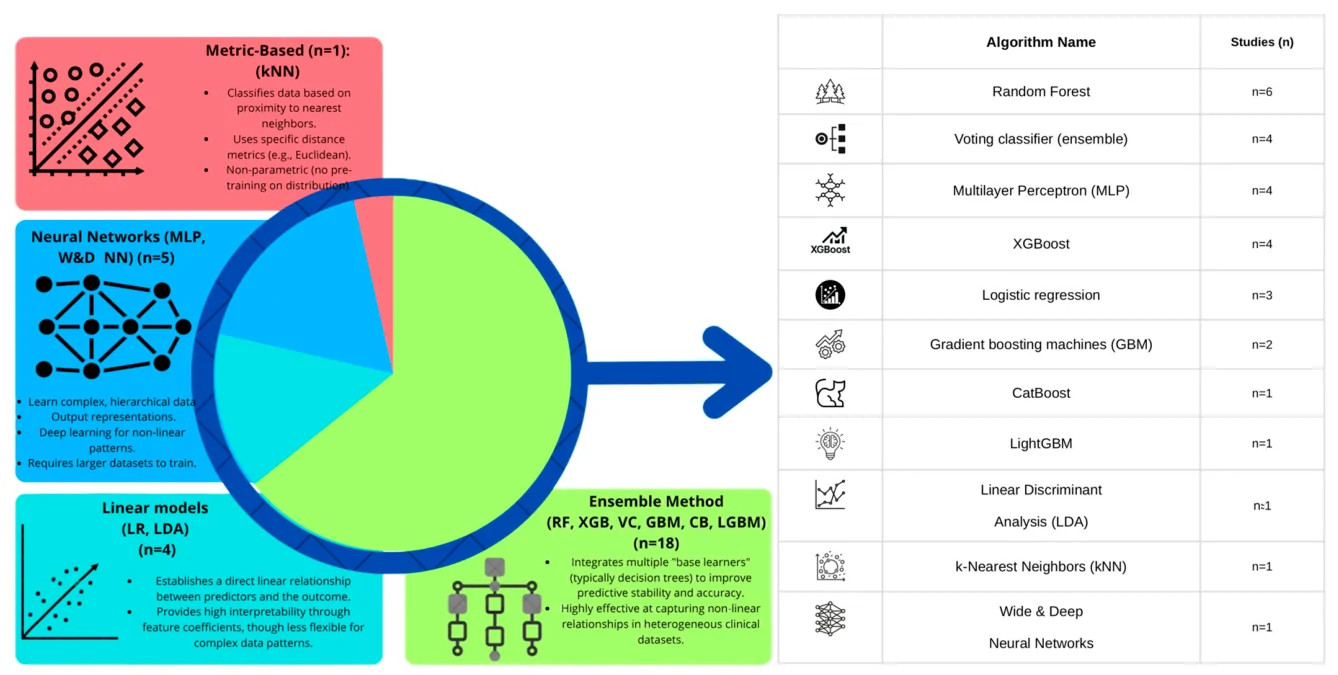
Distribution and characterization of machine learning model architectures identified across included studies. The left panel presents a taxonomic classification of models by algorithmic family: Ensemble Methods (RF, XGB, VC, GBM, CB, LGBM; n = 18), Neural Networks (MLP, W&D NN; n = 5), Linear Models (LR, LDA; n = 4), and Metric-Based Methods (kNN; n = 1), each accompanied by its principal technical characteristics. The central pie chart illustrates the relative proportion of each algorithmic family across the included studies. The right panel disaggregates the most frequently reported individual algorithms: Random Forest (n = 6) [15,17,31,32,34,36], voting classifiers (n = 4) [16,21,26,28], multilayer perceptron (n = 4) [30,33,38,39], XGBoost (n = 4) [18,20,23,40], logistic regression (n = 3) [24,29,37], gradient boosting machines (n = 2) [19,25], and five algorithms represented by a single study each: CatBoost [22], LightGBM [41], linear discriminant analysis [42], k-nearest neighbors [35], and wide and deep neural networks [27].

Random Forest was the most frequently employed approach, appearing in six studies [15,17,31,32,34,36]. XGBoost was used in four studies [18,20,23,40], while multilayer perceptron (MLP) [30,33,38,39] networks and voting classifiers (including ensemble approaches) were each used in four studies [16,21,26,28]. Logistic regression [24,29,37] appeared in three studies, and gradient boosting machines (GBM) in two studies [19,25]. The remaining algorithms, CatBoost [22], LightGBM [41], linear discriminant analysis (LDA) [42], k-nearest neighbors (kNN) [35], and wide and deep neural networks [27] were each represented by a single study. The distribution and characterization of machine learning models are presented in Figure 2.

### 3.4. Features Used in Machine Learning Algorithms for the Detection of Sarcopenia

Regarding the features used for sarcopenia classification, clinical tabular data were the predominant input modality, used across 20 studies [15,16,17,18,19,20,22,23,24,25,26,27,29,32,36,37,41], of which 17 relied exclusively on structured clinical variables and 3 combined tabular data with an additional modality: ultrasound imaging [38], video-based kinematic gait features [39], or plantar pressure signals [21] (Table 2). Most of these studies relied on conventional sociodemographic and anthropometric variables (such as age, sex, BMI, and calf circumference) combined with laboratory biomarkers (albumin, triglycerides, creatinine) and comorbidity indicators (diabetes mellitus, hypertension). Within this category, notable subtypes were identified: one study used functional performance test scores (e.g., Timed Up and Go, Six-Meter Walk, Chair Stand test) as tabular inputs [39], and one study derived its features from chest X-ray predictions of appendicular lean mass and handgrip strength, which were then treated as structured tabular variables [32].

Wearable and physiological signal data constituted the second most common input type, in five studies [28,30,33,35,42], including surface electromyography (sEMG) [28], stimulated muscle contraction signals obtained via neuromuscular electrical stimulation [30,33], radial blood pressure waveforms combined with ECG [42], and inertial measurement unit (IMU)-based acceleration recordings [35]. Gait signal data were used in three studies, captured either via smartphone applications or smart insoles measuring joint angles during walking [31,34] or via sensor fusion systems combining IMU and computer vision signals [40]. Finally, three studies adopted a multimodal approach, combining clinical tabular data with at least one unstructured data source, such as raw ultrasound imaging of the rectus femoris [38], plantar pressure measurements from pressure plates [21], or video-based kinematic gait features [39].

### 3.5. Classification Performance of Machine Learning Models for Sarcopenia

**Figure 3:**
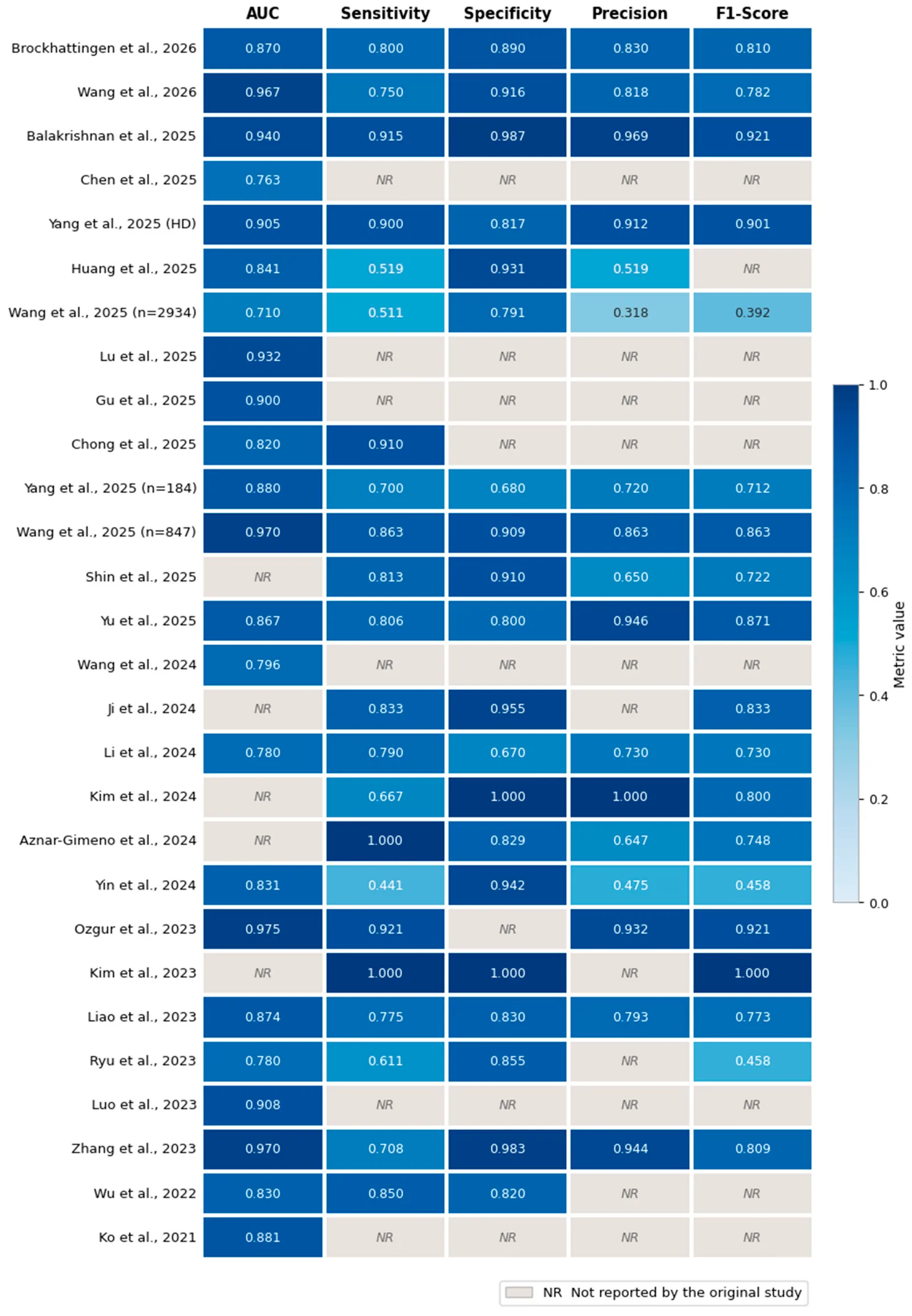
Performance metrics heatmap of machine learning models for sarcopenia detection across included studies [15,16,17,18,19,20,21,22,23,24,25,26,27,28,29,30,31,32,33,34,35,36,37,38,39,40,41,42]. Studies are ordered chronologically from most recent (top) to oldest (bottom). Five performance metrics are displayed where available: AUC, sensitivity, specificity, precision, and F1-score. Colour intensity encodes the metric value on a single-hue sequential scale spanning the full range from 0.0 to 1.0; the scale is not truncated, so that visual differences between cells are proportional to the actual differences between values. Grey cells denote metrics not reported by the original study (NR). For each study, the best-performing model on the overall sample, as designated by the original authors, was used as the unit of extraction. When a metric was reported only stratified by subgroup (e.g., by sex, or by temporal validation cohort), the value of the subgroup reported first by the original authors was used, a rule independent of the magnitude of the estimate. When a study reported both a single hold-out result and a cross-validated result for the same final model, the cross-validated estimate was used. When a study reported performance separately for a validation set and an independent test set, the test set estimate was used. Metrics reported only as ranges across data partitions, or only per diagnostic sub-criterion without a combined value, were classified as NR. Across the 28 studies, AUC was available in 23 studies (range 0.710–0.975), sensitivity in 22 (0.441–1.000), specificity in 20 (0.670–1.000), precision in 17 (0.318–1.000), and F1-score in 19 (0.392–1.000). Reporting was frequently incomplete: 39 of the 140 study-metric combinations were not reported, and six studies reported the AUC as their only performance metric. Perfect specificity (1.000) was reported by two studies (Kim et al., 2023 [34]; Kim et al., 2024 [31]), both with small samples and without external validation; these values should be interpreted as unstable estimates rather than as evidence of perfect classification. Abbreviations: AUC, area under the receiver operating characteristic curve; NR, not reported.

Model performance was reported heterogeneously across studies, with varying completeness across metrics. Classification performance metrics of machine learning models are presented in Figure 3. The AUC was the most consistently reported measure, available in 23 of the 28 included studies, ranging from 0.710 [18] to 0.975 [41]. Given the heterogeneity in study populations, diagnostic criteria, and validation strategies, the mean AUC is reported solely as a descriptive summary (mean = 0.869) and not as a pooled estimate. Nine studies achieved an AUC ≥ 0.90 [15,16,19,20,22,27,37,39,41], nine fell within the 0.80–0.89 range [17,21,23,25,26,29,35,38,42], and five within the 0.70–0.79 range [18,24,28,32,36]. The highest discriminative performance was reported by Ozgur et al. (2023) [41] (LightGBM, AUC = 0.975), Wang et al. (2025) [22] (CatBoost, AUC = 0.970), and Zhang et al. (2023) [27] (Wide and Deep + DNN, AUC = 0.970). However, high AUC values were also reported in studies without external validation and with small sample sizes, such as Balakrishnan et al. (2025) [39], who reported an AUC of 0.940 in a sample of 30 participants without external validation. Additionally, the AUC of 0.967 reported by Wang et al. (2026) [15] was derived from the training set rather than the external validation cohort. The lowest AUC values were observed in studies using large population-based datasets such as CHARLS or NHANES (Wang et al., 2025 [18], AUC = 0.710; Chen et al., 2025 [36], AUC = 0.763).

Considered jointly with study characteristics reported in Table 1, validation strategies in Table 2, and performance metrics in Figure 3, all 13 studies with a sample size below 200 lacked external validation. Among these, two reported AUC values ≥ 0.94: Balakrishnan et al. (2025) [39] (n = 30, AUC = 0.940) and Ozgur et al. (2023) [41] (n = 160, AUC = 0.975, best of three models compared). Among the 15 studies with n ≥ 200, one study without external validation (Wang et al. 2025 [22], n = 847) also reported an AUC ≥ 0.94 (0.970), and one externally validated study (Zhang et al. 2023, n = 4057, external cohort n = 553) reported a comparably high AUC (0.970), indicating that high discriminative performance is not exclusively confined to small, internally validated studies. Wang et al. (2026) [15] (n = 425) also underwent external validation, but the highest AUC reported (0.967) corresponded to the training set rather than the validation cohort. Overall, three of the four studies reporting an AUC ≥0.94 based on a validation estimate did not include external validation.

Sensitivity was reported in 22 studies, ranging from 0.441 to 1.000 (mean = 0.777) [15,16,17,18,21,22,23,25,26,27,28,29,30,31,32,33,38,39,40,41,42], while specificity, available in 20 studies [15,16,17,18,21,22,23,25,26,27,28,30,31,32,33,34,38,39,40,42], ranged from 0.670 to 1.000 (mean = 0.876). F1-score was reported in 19 studies [15,16,18,21,22,23,25,26,27,28,30,31,32,33,34,38,39,40,41] and ranged from 0.392 to 1.000 (mean = 0.763), confirming moderate-to-good overall classification balance across the majority of models.

The prevalence of sarcopenia across the included datasets ranged from 8.3% to 57.3%. Explicit strategies to address this were reported in only 9 of the 28 studies (32.1%) [16,17,21,23,25,26,29,30,38]. The Synthetic Minority Oversampling Technique (SMOTE) was the most common approach (n = 6) [16,21,23,26,29,30], followed by random oversampling (n = 1) [17] and targeted data augmentation of the sarcopenic class (n = 1) [38]; one additional study [25] evaluated combined SMOTE and random undersampling but discarded it after observing no performance gain, retaining an unbalanced final model. A further study [18] applied Youden index threshold optimization, which addresses the decision cut-off rather than the underlying class distribution. The remaining 18 studies (64.3%) did not report any imbalance-handling procedure. None of the studies applying resampling reported a paired comparison of model performance before and after correction.

### 3.6. Calibration and Clinical Utility

Calibration was infrequently reported across the included studies. Only 10 of the 28 studies (35.7%) reported an assessment of model calibration [17,18,19,20,22,23,24,25,32,37], while six studies presented decision curve analysis as a measure of clinical utility [19,20,22,24,25,27].

### 3.7. Adherence to TRIPOD+AI Reporting Items

Adherence to the TRIPOD+AI reporting guideline is presented in Supplementary Table S3. Compliance was evaluated across the 28 included studies using a standardized checklist encompassing 52 items. None of the 28 studies fully satisfied all applicable TRIPOD+AI criteria, indicating that complete compliance with this AI-specific reporting standard remains absent in the current body of machine learning-based sarcopenia prediction research.

The highest level of adherence was observed in the study by Yin et al. (2024), which satisfied 42 of 50 applicable items (84.0%), reflecting strong reporting in core methodological and performance domains but with gaps in areas such as patient involvement and model updating [25]. This was followed by Huang et al. (2025), which met 39 of 48 applicable items (81.2%) [17]. Three studies achieved 38 items each: Chong et al. (2025) (38/46 applicable, 82.6%) [29], Wang et al. (2025) (38/51, 74.5%) [22], and Yu et al. (2025) (38/49, 77.6%) [23].

The items most consistently fulfilled across studies were those related to the classical scientific reporting structure. Specifically, items 1 (Title), 3a–3b (Background), 4 (Objectives), 5a (Data source), 6a–6b (Participants), 8a (Outcome definition), 12e (Analytical methods), 17 (Ethical approval), 21 (Model development), and 26 (Limitations) were reported as fulfilled in all 28 studies. Additionally, items 9a–9b (Predictors), 12a–12c (Analytical methods), 18a (Funding), 18b (Conflicts of interest), 20a–20b (Participants, Results), and 27c (Usability) were fulfilled in 26–27 studies.

In contrast, the items with the lowest compliance reflect critical gaps in transparency, reproducibility, and patient-centered research. Item 18f (Code sharing) remained rare, fulfilled by only two studies: Yin et al., 2024 [25], which provided a public GitHub repository, and Wang et al., 2025 [22], which provided an accessible model repository. Item 19 (Patient and Public Involvement) was not fulfilled by any of the 28 studies. The near absence of publicly available source code, with only 2 of the 28 studies providing a repository and none among other high-scoring studies [17,29], suggests a pervasive culture of limited reproducibility in machine learning-based sarcopenia research, where model performance metrics are prioritized over shareable analytical assets. Similarly, no study reported any form of patient or public involvement in model design, variable selection, or outcome definition, reflecting a broader gap in participatory research practices within the AI-geriatrics field.

Item 8c (Outcome blinding) and item 23b (Model performance across subgroups) were each fulfilled by only one study. The near-universal absence of outcome blinding is largely attributable to the predominance of retrospective designs relying on pre-existing survey or registry datasets such as CHARLS and NHANES, in which blinded outcome adjudication is not applicable by design. The lack of subgroup-stratified performance reporting is particularly concerning, as it prevents assessment of whether models perform equitably across clinically relevant subgroups defined by sex, age, or comorbidity burden.

Items 2 (Structured abstract), 12d (Human–AI comparison), 18c (Protocol availability), and 24 (Model updating) were each fulfilled by only two studies. The low compliance with structured abstract requirements reflects the predominance of journal-specific formatting conventions over TRIPOD+AI standards. The near-total absence of human–AI comparison analyses indicates that most studies focused exclusively on algorithmic benchmarking without evaluating whether model predictions offer added value over clinical judgment alone. Furthermore, the lack of accessible protocols and model updating plans suggests that most investigations were conceived as cross-sectional proof-of-concept studies rather than as steps toward clinical deployment.

Item 18d (Study registration) and item 27a (Usability of the model) were fulfilled by three studies each, and item 14 (Fairness and algorithmic bias assessment) was fulfilled by only five studies. The low rate of fairness evaluation is partly explained by the demographic homogeneity of many study populations, predominantly Chinese cohorts, in which formal algorithmic bias assessment across ethnic or socioeconomic subgroups was neither performed nor considered necessary by the authors. However, even within demographically homogeneous samples, fairness assessment across sex and age strata remains essential and was largely overlooked.

### 3.8. Results of the Methodological Quality Assessment

Overall, the included studies showed variable adherence to individual methodological quality criteria, with consistent strengths in the definition of the research question and study population (C1–C2, 100%), participant selection procedures (C4, 92.9%), and valid and consistently implemented exposure and outcome measures (C9 and C11, 100%). However, important methodological limitations were identified, particularly regarding sample size justification (C5, 21.4%), temporality between exposure and outcome assessment (C6, 7.1%), repeated exposure assessment (C10, 17.9%), and adjustment for potential confounding variables (C14, 25.0%). Participation rates were adequately reported in 28.6% of studies (C3), while blinding of outcome assessors (C12) was not reported. No overall quality score or summary category was derived, as the NHLBI tool does not define validated thresholds for such a classification. Item-level results for all 28 studies are presented in Table 3.

## 4. Discussion

Throughout this section, the terms screening, detection, prediction, and diagnosis are used with the operational definitions given in the Introduction. Two previous reviews have addressed the application of AI to sarcopenia [8,9]; both were broad narrative overviews. Neither restricted eligibility to models performing binary classification against consensus reference standards, nor did either apply a structured appraisal of reporting completeness or methodological quality. The present review adds a formal item-level assessment of adherence to TRIPOD+AI, a standardized appraisal of methodological quality, and an explicit examination of external validation, class imbalance handling, calibration reporting, and code availability. The review aimed to describe the methodological characteristics of these models, the input features used, model performance, validation strategies, and adherence to reporting standards. Our findings indicate that a wide range of machine learning algorithms have been applied to sarcopenia detection, with the most frequently reported models including Random Forest, voting classifiers, and multilayer perceptron (MLP). Overall, these models demonstrated high discriminative performance, with reported areas under the curve (AUC) ranging from 0.710 to 0.975. However, these findings should be interpreted in light of the methodological and reporting limitations of the available evidence. Limitations in sample size justification, control for confounding, validation strategies, and adherence to TRIPOD+AI recommendations may restrict the generalizability and clinical applicability of these models.

The temporal distribution of the included studies shows rapid and recent growth: one study was published in 2021, one in 2022, six in 2023, six in 2024, twelve in 2025, and two in the first weeks of 2026, before the search was closed. Almost half of the available evidence therefore dates from a single year. The algorithms employed also shifted over this period. The two earliest studies applied single classical classifiers (k-nearest neighbors and linear discriminant analysis), whereas from 2023 onward, ensemble methods became dominant, accounting for 18 of the 28 final models overall and for 9 of the 12 models published in 2025. Gradient-boosting variants (XGBoost, LightGBM, CatBoost) appear exclusively from 2023 onward. Input modalities broadened in parallel, with wearable sensors, gait signals, and imaging-derived features appearing alongside clinical tabular data, which nonetheless remained the predominant input type throughout the period. Taken together, these findings show an increase in the number and diversity of models and input modalities over time, whereas external validation remained uncommon throughout the period.

Among the AI approaches identified in this review, ensemble learning methods such as Random Forest and boosting algorithms such as XGBoost were the most frequently applied models. Our findings are consistent with the literature: ensemble methods such as Random Forest [13,43], boosting algorithms such as XGBoost [13], Support Vector Machines [43], Logistic Regression [13,43], and Neural Networks [44] have been identified as the most widely applied approaches in clinical prediction and classification tasks. These algorithms have been widely used in healthcare research for the prediction and classification of various clinical conditions due to their ability to handle complex non-linear relationships and interactions between variables [13,43]. Previous studies applying these models in other medical contexts have also reported high classification performance and robust predictive discrimination [45]. However, few studies included in the present review evaluated whether model predictions improve decision-making compared with standard clinical assessment, highlighting the limited evidence available regarding their added clinical value.

Machine learning models have also been developed for other geriatric syndromes, including frailty [46], cognitive impairment [47], falls [48], and malnutrition [49]. We deliberately refrain from comparing the AUC values reported here with those reported in that literature, because discrimination depends on the outcome definition, the case mix, and the prevalence of the target condition in each cohort, and is therefore not transferable across conditions. What can be compared is the nature of the outcome being modeled, and this comparison is informative. The outcomes themselves differ in how their reference standards are constructed, and that difference bears directly on the modeling task. Sarcopenia is typically defined based on measurable physiological components such as muscle mass, muscle strength, and physical performance, which may generate clearer and more structured patterns within datasets [50]. In contrast, frailty, falls, and cognitive impairment are multidimensional conditions involving physical, psychological, and social domains, which may introduce greater heterogeneity and complexity for predictive modeling [51,52]. For instance, frailty assessment is characterized by substantial methodological variability, with numerous instruments and questionnaires used across studies. These tools often capture different domains of vulnerability and demonstrate only modest agreement with one another, reflecting the absence of a universally accepted operational definition of frailty [53]. Such variability may reduce the consistency of the outcome labels used to train AI models, thereby increasing the difficulty of developing robust and generalizable predictive algorithms. Additionally, current AI studies often benefit from improved computational techniques, larger datasets, and the use of multiple algorithms with cross-validation procedures, which may reduce the optimism of internal performance estimates. Cross-validation, however, resamples the same dataset and therefore addresses overfitting to a particular partition, not transportability to a different population or setting. It should not be taken as evidence of generalizability, which can only be established through external validation [54,55].

Another important methodological limitation concerns model calibration. Discrimination, commonly measured by the AUC, indicates how well a model ranks individuals with and without sarcopenia, but does not determine whether predicted probabilities correspond to observed risks. A model may therefore show high discrimination while remaining poorly calibrated. This distinction is particularly important when predicted probabilities are intended to guide confirmatory assessment or clinical decision-making. In the present review, calibration was assessed in only 10 of the 28 studies, while decision curve analysis was reported in six, indicating that the clinical reliability and potential utility of most models remain insufficiently characterized.

The two lowest AUC values in this review were reported by studies developed in large population-based cohorts (CHARLS and NHANES). One possible explanation is that broad, unselected populations introduce greater case mix heterogeneity than the clinically defined cohorts used in most other included studies, which may reduce apparent discrimination while yielding estimates that are more representative of general population performance. This interpretation is not directly testable with the available data and is offered as a hypothesis rather than as a demonstrated effect.

Regarding the predictors used for sarcopenia classification, most studies included clinical, anthropometric, and sociodemographic variables. This finding is particularly relevant because traditional diagnostic approaches for sarcopenia often require functional assessments such as gait speed or handgrip strength, as well as more advanced techniques to estimate muscle mass, including DXA or BIA [9]. On the other hand, some studies have explored more advanced diagnostic technologies such as electromyography or ultrasound imaging to assess muscle characteristics [56]. Although these techniques can provide detailed physiological information and have demonstrated good diagnostic performance, their accessibility varies considerably. For example, electromyography remains expensive, requires specialized equipment, and is highly operator-dependent, limiting its use in many clinical settings, particularly in low- and middle-income countries [50]. Ultrasound is generally more portable and affordable than DXA, although its application for sarcopenia assessment still requires standardized protocols and trained operators, which remain limited in many settings [57].

In contrast, several studies included in this review reported high discrimination using models that incorporated routinely collected variables, such as calf circumference, body mass index (BMI), and other clinical indicators [19,25,41]. It should be noted, however, that no included study compared models based on routinely collected clinical variables against imaging- or device-based approaches within the same sample. The apparent equivalence between these two strategies therefore rests on indirect, between-study comparison, and should be regarded as a hypothesis requiring head-to-head evaluation rather than as an established finding. The use of these low-cost and easily obtainable variables represents a major advantage, as it allows the development of scalable and accessible screening tools for sarcopenia in primary care and community settings [17,22,27]. From a public health perspective, the integration of AI models based on simple predictors could facilitate earlier identification of individuals at risk of sarcopenia, enabling timely intervention while reducing the need for costly diagnostic procedures.

A notable finding across the included studies was the substantial variability in the way methodological details were reported. The level of detail provided regarding model development, internal validation strategies, handling of missing data, and reproducibility of the modeling pipeline differed markedly between studies. This heterogeneity underscores the importance of adopting standardized reporting frameworks in future AI/ML research on sarcopenia. The TRIPOD+AI statement provides a comprehensive checklist specifically designed for studies developing or evaluating prediction models. Adherence to TRIPOD+AI would promote greater transparency, comparability, and reproducibility across studies, facilitating both critical appraisal by reviewers and meaningful synthesis of evidence. Future investigations developing or validating AI/ML models for sarcopenia should therefore consider TRIPOD+AI from the study design stage onward to improve reporting completeness and facilitate critical appraisal.

### 4.1. Implications for Clinical Practice

The current evidence does not establish the clinical applicability of machine-learning models for sarcopenia classification. The diagnosis of sarcopenia frequently depends on specialized assessments such as dual-energy X-ray absorptiometry (DXA) or functional measurements including gait speed and handgrip strength testing with a dynamometer, which may require specific equipment, trained personnel, and additional time. Machine learning models offer the possibility of screening for sarcopenia using routinely available clinical data, including demographic characteristics, anthropometric measures, and laboratory parameters. By relying on variables that are already collected in many healthcare settings, these approaches may represent a low-cost and scalable strategy for sarcopenia detection. If validated in clinical practice, models with high sensitivity and specificity could facilitate rapid identification of individuals with probable sarcopenia, allowing clinicians to prioritize confirmatory evaluation and initiate timely interventions such as structured exercise programs, nutritional optimization, and targeted rehabilitation. Furthermore, such tools could be particularly valuable in resource-limited settings or healthcare systems without widespread access to geriatric specialists, including primary care environments. In these contexts, machine learning-based prediction models may support earlier recognition of sarcopenia risk and improved allocation of preventive and rehabilitative interventions. At present, however, none of these applications is supported by the available evidence. No included model has been externally validated in a population resembling the intended deployment setting, prospectively evaluated in routine care, or assessed for its effect on clinical decisions or patient outcomes. The clinical potential described above is therefore prospective, and its realization depends on evidence that this body of literature does not yet provide.

### 4.2. Limitations and Future Research

Several limitations of both the available evidence and the present review should be considered when interpreting these findings. First, a considerable proportion of studies did not fully adhere to established reporting guidelines for prediction models based on artificial intelligence, such as the TRIPOD+AI framework, which limits transparency, reproducibility, and the critical appraisal of model development and validation processes. Importantly, none of the included models has been validated as a confirmatory diagnostic test. Across the included studies, AI/ML models were developed either to classify prevalent sarcopenia against a consensus reference standard or, in longitudinal studies, to predict its future occurrence. These applications should therefore be distinguished from confirmatory diagnosis based on established consensus criteria.

Second, a formal statistical sensitivity analysis of the relationship between sample size and AUC was not considered appropriate because AUC estimates were not consistently reported as single, comparable values across studies. Several studies did not report AUC, and at least one high AUC value was derived from the training set rather than an independent validation set. Instead, our descriptive comparison of study characteristics, validation strategies, and reported AUC values showed that all 13 studies with sample sizes < 200 lacked external validation, while three of the four studies reporting a validated AUC ≥ 0.94 also lacked external validation. Conversely, one large, externally validated study [27] reported a comparably high AUC, indicating that high discriminative performance cannot be attributed solely to small sample size or the absence of external validation. These findings warrant caution when interpreting exceptionally high performance estimates and reinforce the need for independent external validation.

Third, most of the studies included in this review were conducted in Asian populations, particularly in China and South Korea, which may limit the geographical and demographic generalizability of the available evidence. External validation was also uncommon, with most studies relying on internal validation methods such as cross-validation or split-sample approaches. While these methods are useful during model development, they do not guarantee that models will maintain their predictive performance when applied to independent populations or different clinical settings. External validation is therefore essential to establish the robustness and generalizability of AI-based models.

Additionally, the included studies applied different diagnostic consensus criteria (AWGS2, EWGSOP2, and FNIH), which use population- and criterion-specific thresholds for muscle mass, strength, and physical performance. Consequently, the reported AUC, sensitivity, specificity, and F1-score are not fully comparable across studies, as they reflect model performance against different reference standards used to define sarcopenia. Accordingly, the descriptive differences observed across diagnostic criteria should not be interpreted as evidence of the superiority of any single consensus definition.

Furthermore, although sarcopenia prevalence varied substantially across cohorts (8.3–57.3%), resulting in heterogeneous degrees of class imbalance, fewer than one-third of the included studies reported strategies to address this issue, and none provided paired comparisons of model performance before and after correction. Consequently, the extent to which reported metrics were influenced by class imbalance management strategies cannot be determined. Although specificity was, on average, higher than sensitivity across the included studies, the available data do not permit determination of whether this pattern resulted from class imbalance, threshold selection, model characteristics, or other study-specific factors.

Transparency and reproducibility were also limited, as only 2 of the 28 studies provided publicly available source code or model repositories. Moreover, substantial heterogeneity in study populations, diagnostic criteria, model architectures, validation strategies, and performance reporting precluded meaningful quantitative synthesis. Performance metrics were inconsistently reported across studies; therefore, standardized extraction rules were applied to enhance comparability. Nevertheless, selecting the best-performing model from each study may have resulted in somewhat optimistic performance estimates. Accordingly, performance measures were summarized descriptively and should not be interpreted as pooled estimates of classification performance.

Limitations were identified in sample size justification, temporality, repeated exposure assessment, and adjustment for potential confounding. Eligibility was restricted to studies published in English, Spanish, or Portuguese, which may have excluded relevant models reported in other languages; this is particularly relevant given the predominance of studies conducted in East Asia. Grey literature, preprints, conference proceedings, and dissertations were not searched, so models reported outside the peer-reviewed journal literature are not represented, and the possibility of publication bias toward studies reporting favorable performance cannot be excluded. Finally, the literature search was completed in February 2026. Given the rapid pace of methodological development in artificial intelligence and machine learning, studies published after this date are not represented in the present review, and the evidence landscape may therefore evolve rapidly.

Future studies should prioritize adequately powered designs, external validation in independent and geographically diverse populations, transparent management of class imbalance and missing data, adherence to TRIPOD+AI, and open sharing of code and model specifications. Future research should also report model calibration, evaluate performance across clinically relevant subgroups, assess fairness and transportability, and prospectively determine whether model-assisted classification provides incremental clinical benefit over existing assessment pathways. These steps are necessary before the clinical implementation of AI/ML models for sarcopenia can be adequately supported.

## 5. Conclusions

Machine learning has been increasingly applied for the detection and prediction of sarcopenia. A wide range of machine learning models has been used, with the most frequently reported algorithms including Random Forest, voting classifiers, and multilayer perceptron (MLP). Reported discrimination was high across the included studies, with AUC values ranging from 0.710 to 0.975. These figures correspond to the best-performing model of each study, derive predominantly from internal validation, and are not comparable across studies, which used different consensus reference standards and populations. They should therefore be read as the performance reported in the literature rather than as an estimate of accuracy in clinical use. The most common predictors incorporated into these models included clinical, anthropometric, and sociodemographic variables. The available evidence showed variable adherence to individual methodological quality criteria, although important methodological limitations remain. Most available studies have been conducted in Asian populations, and external validation was performed in only 7 of the 28 studies. Moreover, no study fully adhered to TRIPOD+AI guidelines for artificial intelligence-based prediction models. The integration of AI-based screening tools into routine clinical workflows would require external validation in the intended target populations, systematic reporting of calibration, assessment of fairness across clinically relevant subgroups, direct comparison against conventional clinical assessment, and prospective evaluation of clinical impact. Each of these remains uncommon in the current evidence base: external validation was performed in 7 of the 28 included studies, calibration was assessed in 10, fairness in 5, and direct comparison with clinical assessment in 2, while no study reported prospective evaluation in routine care. Based on the current evidence, these models should be regarded as promising candidates for case finding rather than tools ready for implementation.

## Figures and Tables

**Table 1:** Characteristics of the study included in the review.

Author (Year)	Country	Study Design	Sample Size (n)	Age (Years)	Female (%)	Clinical Context	Consensus Used to for Sarcopenia	Prevalence (%)
Brockhattingen et al. 2026 [38]	Denmark	Cross-sectional	24	Total = 81 (5.2)	63%	Hospitalized older adults	EWGSOP2	37.5%
Wang et al. 2026 [15]	China	Prospective cohort	425	68.23 ± 6.44	31.4% (non-sarcopenia); 55.9% (sarcopenia)	Hospitalized patients	AWGS2	34.1%
Balakrishnan et al. 2025 [39]	India	Cross-sectional	30	Sarcopenia = 58.6 (15.3) Non-Sarcopenia = 47.9 (15.1)	31.25%	Hospitalized patients	EWGSOP2	NR
Chen et al. 2025 [36]	United States	Cross-sectional	4012	Sarcopenia = 43 (11) Non-Sarcopenia = 39 (12)	49%	Community-dwelling adults	FNIH	8.3%
Yang et al. 2025 [16]	China	Cross-sectional	164	Sarcopenia Total = 60.35 (12.72) Female = 59.56 (12.56) Male = 61.33 (12.99) Non-Sarcopenia Total = 47.70 (9.79) Female = 45.86 (8.59) Male = 49.00 (10.46)	49.39%	Hemodialysis center	AWGS2	57.3%
Huang et al. 2025 [17]	China	Cross-sectional	910	Total = 71.76 ± 5.67	55.8%	Community-dwelling older adults	AWGS2	20.1%
Wang et al. 2025 [18]	China	Prospective cohort	2934	Total = 53	52.6	Community-dwelling older adults	AWGS2	16.7%
Lu et al. 2025 [19]	China	Cross-sectional	1092	Total = 65.41 ± 9.81	50.50%	Community-dwelling older adults	AWGS2	21.2%
Gu et al. 2025 [20]	China	Retrospective cross-sectional	231	Total = 66	23	Hospitalized patients	AWGS2	55.4%
Chong et al. 2025 [29]	Republic of Korea	Cross-sectional	1926 (training/test) + 2247 (external validation)	Total = 40–64	Sarcopenia group: 71.9% female; non-sarcopenia: 56.3% female)	Community-dwelling adults	AWGS2	12.1%
Yang et al. 2025 [21]	China	Cross-sectional	184	Total: 53.66 Female = 53.91 Male = 53.91	48.37	Hospitalized patients	AWGS2	52.7%
Wang et al. 2025 [22]	China	Cross-sectional	847	65 years and above	64.46%	Community-dwelling older adults	AWGS2	NR
Shin et al. 2025 [33]	South Korea	Cross-sectional	199	Total = 74.7 Female = 74.5 Male = 75.0	52.76	Hospitalized patients	AWGS2	20.1%
Yu et al. 2025 [23]	China	Longitudinal cohort (CHARLS 2011–2015)	1080	Total = 66.32 (5.23)	52.50%	Community-dwelling older adults	AWGS2	18.6%
Wang et al. 2024 [24]	China	Cross-sectional	1434	Sarcopenia = 68.23 (6.44) Non-sarcopenia = 68.23 (6.44)	52.4%	Hospitalized patients	AWGS2	39.8%
Ji et al. 2024 [30]	Korea	Cross-sectional	40	Sarcopenia Total = 62 (11.0) Female = 59.0 (7.6) Male = 67.3 (7.9) Non-sarcopenia Total = 62 (11.0) Female = 73.6 (10.5) Male = 56.6 (8.7)	30%	Hospitalized patients	EWGSOP2	25.0%
Li et al. 2024 [28]	China	Cross-sectional	93	Sarcopenia Total = 75.3 (7.7) Female = 73.8 (8.0) Male = 77.9 (6.6) Non-sarcopenia: Total = 68.6 (5.8) Female = 67.7 (5.9) Male = 69.7 (5.6)	69.9%	Community-dwelling older adults	AWGS2	51.6%
Kim et al. 2024 [31]	South Korea	Cross-sectional	38	Male = 65.1 (7.6) Female = 61.1 (8.1)	68.4%	Hospitalized patients	AWGS2	23.7%
Aznar-Gimeno et al. 2024 [40]	Spain	Cross-sectional	42	Total = 70.5 (66–81)	57.14%	Hospitalized patients	EWGSOP2	16.7%
Yin et al. 2024 [25]	China	Cross-sectional	11,661 (baseline); 9403 (2013 follow-up); 10,356 (2015 follow-up)	Total = 57.0	51.7%	Community older adults	AWGS2	11.0%
Ozgur et al. 2023 [41]	Turkey	Cross-sectional	160	Total = NR Female = 72.4 (5.6) Male = 75.4 (6.7)	NR	Community-dwelling older adults	EWGSOP2	NR
Kim et al. 2023 [34]	South Korea	Cross-sectional	83	Sarcopenia: Total = 77.8 (8.6) Female = 77.5 (9.3) Male = 78.4 (7.5) Non-sarcopenia: Total = 63.4 (15.4) Female = 69.0 (16.5) Male = 58.1 (12.4)	59.04%	Hospitalized patients	AWGS2	27.7%
Liao et al. 2023 [26]	China	Cross-sectional	242	Sarcopenia Total = NR Female = 57.28 (14.23) Male = 61.00 (12.23) Non-sarcopenia Total = NR Female = 48.57 (10.18) Male = 45.90 (11.04)	42.1%	Hospitalized patients	AWGS2	50.8%
Ryu et al. 2023 [32]	South Korea	Prospective cohort with external validation	1075	Total = 61.4	76%	Hospitalized patients	AWGS2	15%
Luo et al. 2023 [37]	United States	Prospective cohort	1304	Sarcopenia = 58.36 (16.1) Non-sarcopenia = 52.13 (14.7)	79.8%	Hospitalized patients	EWGSOP2	19.1%
Zhang et al. 2023 [27]	China	Cross-sectional	4057 (training/test) + 553 (external validation)	Sarcopenia = 67.89 (8.66) Non-sarcopenia = 61.01 (7.54).	NR	Community-dwelling	AWGS2	19.0%
Wu et al. 2022 [42]	Taiwan	Cross-sectional	133	Total = 71.01 (4.14) Sarcopenia = 14 (10.5) Presarcopenia = 17 (12,8) Possible Sarcopenia = 59 (44.4)	59.9	Hospitalized patients	AWGS2	44.4%
Ko et al. 2021 [35]	South Korea	Cross-sectional	78	Total = 78.8 (5.7)	100%	Community-dwelling	AWGS2	25.6%

Abbreviations: AWGS2, Asian Working Group for Sarcopenia 2019 consensus; EWGSOP2, European Working Group on Sarcopenia in Older People 2; FNIH, Foundation for the National Institutes of Health Sarcopenia Project; CHARLS, China Health and Retirement Longitudinal Study; NR, not reported. Notes: Age is reported as presented in the original studies (mean ± standard deviation, mean [standard deviation], median [interquartile range], or range, as applicable). Sample sizes include external validation cohorts when these were reported separately. Percentages correspond to the information reported by the original studies and may refer to the total sample or specific study groups, as indicated in the table.

**Table 2:** Model Architecture, Input Features, Validation Strategy, and Output Format of Machine Learning Algorithms for Sarcopenia detection.

Author and Year	Model	Features (Standardized)	Feature Selection	Missing Data Management	Validation	External Validation	Output Format
Brockhattingen et al. 2026 [38]	Multilayer Perceptron Network	Combined: Clinical tabular data (age, gender, height, weight, BMI, SPPB score) + imaging data (longitudinal and transverse ultrasound images of the rectus femoris)	No	No reported	k-fold cross-validation	No	Binary classification
Wang et al. 2026 [15]	RF	Clinical tabular data (BMI, serum albumin, age, uric acid, creatinine, hemoglobin, calcium, triglycerides, CRP, total protein, urea, NIHSS score)	RF algorithm (importance threshold > 0.031)	No reported	Hold-out external (stratified sampling: 280 training, 145 validation)	Yes	Binary classification
Balakrishnan et al. 2025 [39]	MLP	Combined: Clinical tabular data (functional performance tests: TUG, Six-Metre Walk, Chair Stand, Body Composition) + gait signal data (kinematic features from video-based gait analysis)	No reported	No reported	5-fold cross-validation	No	Binary classification
Chen et al. 2025 [36]	RF	Clinical tabular data (ZJU index, FPG, BMI, ALT/AST ratio, TG, age, gender, ethnicity, education, marital status, poverty-income ratio, tobacco use, alcohol use, diabetes, hypertension, total cholesterol, frailty, nutritional status, physical activity, osteoporosis)	Boruta algorithm, SHAP	Participants with missing data were excluded	Hold-out (test set)	No	Binary classification
Yang et al. 2025 [16]	Voting classifier	Clinical tabular data (hip/knee/ankle joint force, handgrip strength, body measurements, basic information, laboratory findings)	Lasso regression, importance scores ranking	Interpolation using mean of corresponding feature from instances with matching gender and sarcopenia status	Stratified shuffling	No	Binary classification
Huang et al. 2025 [17]	RF	Clinical tabular data (weight, age, calf circumference, triglycerides)	LASSO regression with 10-fold cross-validation	Multiple imputation	Hold-out split 8:2 + 10-fold CV	Yes,	Binary classification
Wang et al. 2025 [18]	XGBoost	Clinical tabular data (demographics, lifestyle, health status questionnaire + blood biomarkers)	Two-step: univariate analysis + regularization (LASSO-type)	Multiple imputation	Hold-out split 8:2 + 5-fold CV	Yes	Binary classification
Lu et al. 2025 [19]	GBM	Clinical tabular data (sociodemographic, behavioral, health status variables: waist circumference, diastolic blood pressure; biochemical: LDL-C, HDL-C, triglycerides)	LASSO	Participants with missing data were excluded	Hold-out split 7:3	No	Nomogram + ML model (GBM)
Gu et al. 2025 [20]	XGBoost	Clinical tabular data (perioperative: serum albumin, diabetes, operation style, nutritional score NRS-2002, ECOG performance status)	LASSO regression + Venn diagram (cross-validated with univariate analysis)	No reported	Hold-out split	No	Binary classification
Chong et al. 2025 [29]	Logistic Regression	Clinical tabular data (age, gender, education, marital status, BMI, hypertension, diabetes, hypercholesterolemia, anemia, hs-CRP, comorbidities, smoking, alcohol, sitting time, protein intake, aerobic/strength exercise, perceived stress, GAD-7 score)	L1-regularized logistic regression, recursive feature elimination	Participants with missing data were excluded	5-fold stratified cross-validation, Hold-out 75/25	Yes	Binary classification
Yang et al. 2025 [21]	Voting classifier	Combined: Clinical tabular data (age, pre-dialysis creatinine) + wearable signal data (plantar pressure biomechanical indicators from pressure plate)	Statistical analysis + feature importance evaluation with Lasso	Mean imputation using comparable instances with same gender and sarcopenia condition	Stratified shuffling split	No	Binary classification
Wang et al. 2025 [22]	CatBoost	Clinical tabular data (95 variables from CHARLS: demographics, lifestyle, health status, laboratory values)	LASSO, logistic regression	Participants with missing data were excluded	10-fold Cross-validation, Hold-out 7:3	No	Binary classification
Shin et al. 2025 [33]	MLP	Wearable signal data (stimulated muscle contraction signals via neuromuscular electrical stimulation, exoPill device)	mRMR	Not applicable	Nested 3 × 2 cross-validation	No	Binary classification
Yu et al. 2025 [23]	XGBoost	Clinical tabular data (BMI, height, age, DBP, lipids: TG/TC/LDL, glucose, BUN, HDL-C, uric acid, CES-D depression score, smoking, alcohol, physical activity, diet)	Univariate logistic regression + collinearity filtering (r > 0.90)	Multiple imputation	Hold out 7:3; 5-fold CV for hyperparameter tuning; 100 random seed iterations for robustness	No	Binary classification + risk stratification scores
Wang et al. 2024 [24]	Logistic Regression	Clinical tabular data (age, BMI, diabetic duration, HbA1c, 25-OH Vitamin D, nephropathy, neuropathy, nutritional status, osteoporosis)	Least Absolute Shrinkage and Selection Operator (LASSO)	No reported	Hold-out 70:30	No	Nomograme
Ji et al. 2024 [30]	MLP	Wearable signal data (stimulated muscle contraction signals from wearable device)	Whitney test, *p* < 0.01	Not applicable	Leave-one-out cross-validation (LOOCV)	No	Binary classification
Li et al. 2024 [28]	Voting classifier	Wearable signal data (surface electromyography signals from forearm, sEMG)	No	Not applicable	5-fold cross-validation	No	Binary classification
Kim et al. 2024 [31]	RF	Gait signal data (joint angle measurements during walking, smart insole)	Gini importance	Not applicable	Hold-out 75/25 with 10 iterations	No	Binary classification
Aznar-Gimeno et al. 2024 [40]	XGBoost	Gait signal data (step characteristics, joint angles, temporal-spatial gait parameters from combined IMU and computer vision sensor fusion)	Shapley values along with the XGBoost algorithm	Not applicable	5-fold cross-validation	No	Binary classification
Yin et al. 2024 [25]	GBM	Clinical tabular data (functional performance tests: age, sex, height, weight + 20 binary functional capacity items)	Hypothesis-driven (20 FC items pre-specified) + age, sex, height, weight included a priori; threshold optimization applied	Participants with missing data were excluded	10-fold cross-validation + hold-out 7:3	Yes	Binary classification
Ozgur et al. 2023 [41]	LightGBM	Clinical tabular data (sex, age, BMI, hypertension, diabetes, SARC-F score, MNA score, calf circumference, gait speed, handgrip strength, mid-upper arm circumference)	Statistical tests	No reported	Hold-out 80/20	No	Binary classification
Kim et al. 2023 [34]	RF	Gait signal data (joint angle measurements during walking, smartphone application)	MeanDecreaseGini coefficient	Not applicable	Hold-out 67/33	No	Binary classification
Liao et al. 2023 [26]	Voting classifier	Clinical tabular data (age, sex, anthropometric parameters, handgrip strength, laboratory biomarkers, body composition by BIA, dialysis-related variables)	Least Absolute Shrinkage and Selection Operator (LASSO)	Participants with missing data were excluded	Hold-out split	No	Binary classification
Ryu et al. 2023 [32]	Random Forest	Clinical tabular data (image-derived features: age, sex, BMI + predicted ALM, handgrip strength and chair rise test performance from chest X-ray, with estimation uncertainty values)	No	Participants with missing data were excluded	Hold-out (65% train, 15% validation, 20%) with three-fold five-repeat cross-validation for hyperparameter tuning	Yes	SARC-CXR Score (continuous score for classification of sarcopenia presence)
Luo et al. 2023 [37]	Logistic Regression	Clinical tabular data (EHR-derived: 206 diagnosis categories, 67 laboratory variables, 71 medication variables, BMI)	SHAP (SHapley Additive exPlanations) with elbow method	Participants with missing data were excluded	10-fold cross-validation on training data; 70% training, 10% validation, 20% test set split	No	Binary classification (probability)
Zhang et al. 2023 [27]	W&D + DNN	Clinical tabular data (age, weight, triceps skinfold thickness, calf circumference, mid-upper arm circumference, AST/ALT ratio, physical activity)	Principal Component Analysis (PCA) from 109 original features	Participants with missing data were excluded	5-fold stratified cross-validation, Hold-out 8:2	Yes	Binary classification
Wu et al. 2022 [42]	Self-developed scoring system among LDA	Wearable signal data (noninvasive radial blood pressure waveform + ECG)	Selection based on significant or marginally significant differences (0.05 < *p* < 0.1) between possible-sarcopenia and robust subjects	Not applicable	Threefold cross-validation	No	Binary classification
Ko et al. 2021 [35]	kNN	Wearable signal data (IMU-based acceleration and angular velocity signals)	Kruskal–Wallis test	Participants with missing data were excluded	NR	No	Binary classification

Abbreviations: ALM, appendicular lean mass; BIA, bioelectrical impedance analysis; BMI, body mass index; BUN, blood urea nitrogen; CES-D, Center for Epidemiologic Studies Depression Scale; CRP, C-reactive protein; CV, cross-validation; DBP, diastolic blood pressure; DNN, deep neural network; ECG, electrocardiography; ECOG, Eastern Cooperative Oncology Group; EHR, electronic health record; GBM, gradient boosting machine; HDL-C, high-density lipoprotein cholesterol; IMU, inertial measurement unit; kNN, k-nearest neighbors; LASSO, Least Absolute Shrinkage and Selection Operator; LDA, linear discriminant analysis; LDL-C, low-density lipoprotein cholesterol; LightGBM, Light Gradient Boosting Machine; LOOCV, leave-one-out cross-validation; MLP, multilayer perceptron; MNA, Mini Nutritional Assessment; mRMR, minimum redundancy maximum relevance; NIHSS, National Institutes of Health Stroke Scale; NR, not reported; PCA, principal component analysis; RF, Random Forest; SARC-F, Strength, Assistance with walking, Rise from a chair, Climb stairs, and Falls questionnaire; sEMG, surface electromyography; SHAP, SHapley Additive exPlanations; SPPB, Short Physical Performance Battery; TG, triglycerides; TUG, Timed Up and Go; W&D, wide and deep; XGBoost, Extreme Gradient Boosting.

**Table 3:** Methodological quality assessment of included studies.

Author (Year)	C1	C2	C3	C4	C5	C6	C7	C8	C9	C10	C11	C12	C13	C14
Brockhattingen et al. 2026 [38]	Y	Y	NR	NR	N	N	N	Y	Y	N	Y	NR	NA	N
Wang et al. 2026 [15]	Y	Y	Y	Y	N	N	N	Y	Y	N	Y	NR	NA	N
Balakrishnan et al. 2025 [39]	Y	Y	N	Y	N	N	N	Y	Y	N	Y	NR	NA	N
Chen et al. 2025 [36]	Y	Y	Y	Y	Y	N	N	Y	Y	N	Y	NR	NA	Y
Yang et al. 2025 [16]	Y	Y	Y	Y	Y	N	NA	Y	Y	N	Y	NR	NA	N
Huang et al. 2025 [17]	Y	Y	NR	Y	Y	N	N	Y	Y	N	Y	NR	NA	N
Wang et al. 2025 [18]	Y	Y	Y	Y	N	N	NA	Y	Y	N	Y	NR	NA	N
Lu et al. 2025 [19]	Y	Y	N	Y	N	N	NA	Y	Y	N	Y	NR	NA	Y
Gu et al. 2025 [20]	Y	Y	NR	Y	N	Y	Y	Y	Y	N	Y	NR	NA	N
Chong et al. 2025 [29]	Y	Y	Y	Y	N	N	N	Y	Y	N	Y	NR	NA	Y
Yang et al. 2025 [21]	Y	Y	NR	Y	N	N	NA	Y	Y	N	Y	NR	NA	N
Wang et al. 2025 [22]	Y	Y	NR	Y	N	N	NA	Y	Y	N	Y	NR	NA	Y
Shin et al. 2025 [33]	Y	Y	NR	Y	N	N	N	Y	Y	N	Y	NR	NA	N
Yu et al. 2025 [23]	Y	Y	NR	Y	N	Y	Y	Y	Y	N	Y	NR	NA	Y
Wang et al. 2024 [24]	Y	Y	Y	Y	Y	N	N	Y	Y	N	Y	NR	NA	Y
Ji et al. 2024 [30]	Y	Y	NR	Y	N	N	N	Y	Y	Y	Y	NR	NA	N
Li et al. 2024 [28]	Y	Y	NR	Y	N	N	N	Y	Y	Y	Y	NR	NA	N
Kim et al. 2024 [31]	Y	Y	NR	Y	N	N	NA	Y	Y	N	Y	NR	NA	N
Aznar-Gimeno et al. 2024 [40]	Y	Y	NR	Y	N	N	NA	Y	Y	N	Y	NR	NA	N
Yin et al. 2024 [25]	Y	Y	NR	Y	N	N	Y	Y	Y	Y	Y	NR	NA	N
Ozgur et al. 2023 [41]	Y	Y	NR	Y	Y	N	N	Y	Y	Y	Y	NR	NA	N
Kim et al. 2023 [34]	Y	Y	NR	NR	N	N	N	Y	Y	N	Y	NR	NA	N
Liao et al. 2023 [26]	Y	Y	NR	Y	N	N	N	Y	Y	N	Y	NR	NA	N
Ryu et al. 2023 [32]	Y	Y	Y	Y	N	N	NA	NA	Y	N	Y	NR	NA	Y
Luo et al. 2023 [37]	Y	Y	NR	Y	Y	N	N	Y	Y	N	Y	NR	NA	N
Zhang et al. 2023 [27]	Y	Y	Y	Y	N	N	NA	Y	Y	N	Y	NR	NA	N
Wu et al. 2022 [42]	Y	Y	NR	Y	N	N	NA	Y	Y	N	Y	NR	NA	N
Ko et al. 2021 [35]	Y	Y	NR	Y	N	N	N	Y	Y	Y	Y	NR	NA	N

Note: C1–C14 correspond to the 14 criteria of the NHLBI Quality Assessment Tool for Observational Cohort and Cross-Sectional Studies. Each study was evaluated according to the information reported in the original publication. C1, Was the research question or objective in this paper clearly stated?; C2, Was the study population clearly specified and defined?; C3, Was the participation rate of eligible persons at least 50%?; C4, Were all subjects selected or recruited from the same or similar populations, including the same time period, and were pre-specified inclusion and exclusion criteria applied uniformly to all participants?; C5, Was a sample size justification, power description, or variance and effect estimate provided?; C6, Were the exposure(s) of interest measured prior to the outcome(s) being measured?; C7, Was the timeframe sufficient to reasonably expect an association between exposure and outcome, if one existed?; C8, For exposures that can vary in amount or level, did the study examine different levels of exposure as related to the outcome?; C9, Were the exposure measures clearly defined, valid, reliable, and implemented consistently across all study participants?; C10, Was the exposure assessed more than once over time?; C11, Were the outcome measures clearly defined, valid, reliable, and implemented consistently across all study participants?; C12, Were the outcome assessors blinded to the exposure status of participants?; C13, Was loss to follow-up after baseline 20% or less?; C14, Were key potential confounding variables measured and adjusted statistically for their impact on the relationship between exposure(s) and outcome(s). Y = Yes; N = No; NR = Not Reported; NA = Not Applicable.

## Data Availability

The data analyzed in this scoping review were extracted from previously published studies. All data supporting the findings are presented in the manuscript and its Supplementary Materials.

## Supplementary Materials

The following supporting information can be downloaded at https://www.mdpi.com/article/10.3390/jcm15218320/s1, Table S1. Search strategy; Table S2. Diagnostic performance of machine learning models for sarcopenia detection, stratified by diagnostic consensus criterion; Table S3. TRIPOD+AI Reporting Compliance Across Included Studies on Machine Learning Models for Sarcopenia Diagnosis. PRISMA 2020 checklist.

## Abbreviations

The following abbreviations are used in this manuscript:

AI	Artificial Intelligence
AUC	Area Under the Curve
AWGS	Asian Working Group for Sarcopenia
AWGS2	Asian Working Group for Sarcopenia 2019 Consensus
BIA	Bioelectrical Impedance Analysis
CHARLS	China Health and Retirement Longitudinal Study
CRP	C-Reactive Protein
CT	Computed Tomography
CV	Cross-Validation
DeCS	Health Sciences Descriptors (Descriptores en Ciencias de la Salud)
DNN	Deep Neural Network
DXA	Dual-Energy X-ray Absorptiometry
EHR	Electronic Health Record
EWGSOP2	European Working Group on Sarcopenia in Older People 2
FNIH	Foundation for the National Institutes of Health
GBM	Gradient Boosting Machine
GLIS	Global Leadership Initiative on Sarcopenia
IMU	Inertial Measurement Unit
kNN	k-Nearest Neighbors
LASSO	Least Absolute Shrinkage and Selection Operator
LDA	Linear Discriminant Analysis
LightGBM	Light Gradient Boosting Machine
ML	Machine Learning
MLP	Multilayer Perceptron
MRI	Magnetic Resonance Imaging
NR	Not Reported
OSF	Open Science Framework
PCA	Principal Component Analysis
PRISMA	Preferred Reporting Items for Systematic Reviews and Meta-Analyses
PRISMA-ScR	Preferred Reporting Items for Systematic Reviews and Meta-Analyses Extension for Scoping Reviews
RF	Random Forest
SHAP	SHapley Additive exPlanations
SPPB	Short Physical Performance Battery
TRIPOD+AI	Transparent Reporting of a Multivariable Prediction Model for Individual Prognosis or Diagnosis + Artificial Intelligence
XGBoost	Extreme Gradient Boosting

## References

[1] Cruz-Jentoft A.J., Bahat G., Bauer J., Boirie Y., Bruyère O., Cederholm T., Cooper C., Landi F., Rolland Y., Sayer A.A. (2019). Sarcopenia: Revised European Consensus on Definition and Diagnosis. Age Ageing.

[2] Álvarez-Bustos A., Rodríguez-Sánchez B., Carnicero-Carreño J.A., Sepúlveda-Loyola W., Garcia-Garcia F.J., Rodríguez-Mañas L. (2022). Healthcare Cost Expenditures Associated to Frailty and Sarcopenia. BMC Geriatr..

[3] Chen L.-K., Woo J., Assantachai P., Auyeung T.-W., Chou M.-Y., Iijima K., Jang H.C., Kang L., Kim M., Kim S. (2020). Asian Working Group for Sarcopenia: 2019 Consensus Update on Sarcopenia Diagnosis and Treatment. J. Am. Med. Dir. Assoc..

[4] Studenski S.A., Peters K.W., Alley D.E., Cawthon P.M., McLean R.R., Harris T.B., Ferrucci L., Guralnik J.M., Fragala M.S., Kenny A.M. (2014). The FNIH Sarcopenia Project: Rationale, Study Description, Conference Recommendations, and Final Estimates. J. Gerontol. A. Biol. Sci. Med. Sci..

[5] Kirk B., Cawthon P.M., Arai H., Ávila-Funes J.A., Barazzoni R., Bhasin S., Binder E.F., Bruyère O., Cederholm T., Chen L.-K. (2024). An Executive Summary on the Global Conceptual Definition of Sarcopenia. Aging Clin. Exp. Res..

[6] Phu S., Vogrin S., Zanker J., Bani Hassan E., Al Saedi A., Duque G. (2019). Agreement Between Initial and Revised European Working Group on Sarcopenia in Older People Definitions. J. Am. Med. Dir. Assoc..

[7] Bruyère O., Beaudart C., Reginster J.-Y., Buckinx F., Schoene D., Hirani V., Cooper C., Kanis J.A., Rizzoli R., McCloskey E. (2016). Assessment of Muscle Mass, Muscle Strength and Physical Performance in Clinical Practice: An International Survey. Eur. Geriatr. Med..

[8] Mustapoevich D.T., Kim W. (2023). Machine Learning Applications in Sarcopenia Detection and Management: A Comprehensive Survey. Healthcare.

[9] Yousaf M.W., Nadeem A.H., Nadeem M.F., Qaisar R. (2026). The Role of Artificial Intelligence in Sarcopenia: Advances, Applications, and Future Directions. Comput. Biol. Chem..

[10] Peters M.D.J., Marnie C., Tricco A.C., Pollock D., Munn Z., Alexander L., McInerney P., Godfrey C.M., Khalil H. (2020). Updated Methodological Guidance for the Conduct of Scoping Reviews. JBI Evid. Synth..

[11] Tricco A.C., Lillie E., Zarin W., O’Brien K.K., Colquhoun H., Levac D., Moher D., Peters M.D.J., Horsley T., Weeks L. (2018). PRISMA Extension for Scoping Reviews (PRISMA-ScR): Checklist and Explanation. Ann. Intern. Med..

[12] Collins G.S., Moons K.G.M., Dhiman P., Riley R.D., Beam A.L., Van Calster B., Ghassemi M., Liu X., Reitsma J.B., van Smeden M. (2024). TRIPOD+AI Statement: Updated Guidance for Reporting Clinical Prediction Models That Use Regression or Machine Learning Methods. BMJ.

[13] Nwanosike E.M., Conway B.R., Merchant H.A., Hasan S.S. (2022). Potential Applications and Performance of Machine Learning Techniques and Algorithms in Clinical Practice: A Systematic Review. Int. J. Med. Inf..

[14] Moons K.G.M., Damen J.A.A., Kaul T., Hooft L., Andaur Navarro C., Dhiman P., Beam A.L., Van Calster B., Celi L.A., Denaxas S. (2025). PROBAST+AI: An Updated Quality, Risk of Bias, and Applicability Assessment Tool for Prediction Models Using Regression or Artificial Intelligence Methods. BMJ.

[15] Wang J., Mu M., Zhang J., Yin X., Gao Y. (2026). Development of Machine Learning-Based Models for Predicting Sarcopenia Risk in Stroke Patients and Analysis of Associated Factors. Sci. Rep..

[16] Yang Y., Liao H., Chen Y., Qiu Y., Yan F., Fu P., Yue J., Chen Y., Yuan H. (2025). Gender-Specific Sarcopenia Screening in Hemodialysis: Insights from Lower Limb Strength and Physiological Indicators. BMC Nephrol..

[17] Huang H., Jiang S., Chen Z., Yu X., Ren K., Zhao Q. (2025). A W-ACT Model for Sarcopenia among Community-Dwelling Older Adults Based on National Basic Public Health Services: Development and Validation Study. Front. Public Health.

[18] Wang Z., Wu Y., Zhu J., Fang Y. (2025). Machine Learning-Based Prediction of Sarcopenia in Community-Dwelling Middle-Aged and Older Adults: Findings from the CHARLS. Psychogeriatrics.

[19] Lu R., Wang S., Chen P., Li F., Li P., Chen Q., Li X., Li F., Guo S., Zhang J. (2025). Predictive Model for Sarcopenia in Chronic Kidney Disease: A Nomogram and Machine Learning Approach Using CHARLS Data. Front. Med..

[20] Gu Y., Su S., Wang X., Mao J., Ni X., Li A., Liang Y., Zeng X. (2025). Comparative Study of XGBoost and Logistic Regression for Predicting Sarcopenia in Postsurgical Gastric Cancer Patients. Sci. Rep..

[21] Yang Y., Liao H., Chen Y., Qiu Y., Yan F., Fu P., Yue J., Chen Y., Yuan H. (2025). Plantar Pressure Distribution Can Be Used to Identify Sarcopenia in Maintenance Hemodialysis Patients. Sci. Rep..

[22] Wang Z., Zhou Y., Zeng X., Zhou Y., Yang T., Hu K. (2025). An Explainable Machine Learning-Based Prediction Model for Sarcopenia in Elderly Chinese People with Knee Osteoarthritis. Aging Clin. Exp. Res..

[23] Yu P., Zhang X., Sun G., Zeng P., Zheng C., Wang K. (2025). Sarcopenia Prediction Model Based on Machine Learning and SHAP Values for Community-Based Older Adults with Cardiovascular Disease in China. Front. Public Health.

[24] Wang X., Gao S. (2024). Development and Validation of a Risk Prediction Model for Sarcopenia in Chinese Older Patients with Type 2 Diabetes Mellitus. Diabetes Metab. Syndr. Obes..

[25] Yin L., Zhao J. (2024). An Artificial Intelligence Approach for Test-Free Identification of Sarcopenia. J. Cachexia Sarcopenia Muscle.

[26] Liao H., Yang Y., Zeng Y., Qiu Y., Chen Y., Zhu L., Fu P., Yan F., Chen Y., Yuan H. (2023). Use Machine Learning to Help Identify Possible Sarcopenia Cases in Maintenance Hemodialysis Patients. BMC Nephrol..

[27] Zhang H., Yin M., Liu Q., Ding F., Hou L., Deng Y., Cui T., Han Y., Pang W., Ye W. (2023). Machine and Deep Learning-Based Clinical Characteristics and Laboratory Markers for the Prediction of Sarcopenia. Chin. Med. J..

[28] Li N., Ou J., He H., He J., Zhang L., Peng Z., Zhong J., Jiang N. (2024). Exploration of a Machine Learning Approach for Diagnosing Sarcopenia among Chinese Community-Dwelling Older Adults Using sEMG-Based Data. J. Neuroeng. Rehabil..

[29] Chong H.J. (2025). A Machine Learning Model for Predicting Sarcopenia Among Middle-Aged Adults: Development and External Validation. JMIR Med. Inform..

[30] Ji Y., Yoon M.-J., Song K., Choi S., Lee H., Jung J.Y., Song S., Kim I., Kim J.Y., Im S. (2024). Feasibility of Sarcopenia Diagnosis Using Stimulated Muscle Contraction Signal in Hemiplegic Stroke Patients. Brain Neurorehabil.

[31] Kim M., Kim D., Kang H., Park S., Kim S., Yoo J.-I. (2024). A Machine Learning Model for Prediction of Sarcopenia in Patients with Parkinson’s Disease. PLoS ONE.

[32] Ryu J., Eom S., Kim H.C., Kim C.O., Rhee Y., You S.C., Hong N. (2023). Chest X-Ray-Based Opportunistic Screening of Sarcopenia Using Deep Learning. J. Cachexia Sarcopenia Muscle.

[33] Shin J., Song K., Kim S.-W., Choi S., Lee H., Kim I.-S., Im S., Baek M.S. (2025). A Wearable Approach for Sarcopenia Diagnosis Using Stimulated Muscle Contraction Signal. Biomed. Eng. Lett..

[34] Kim S., Kim H., Yoo J. (2023). Sarcopenia Classification Model for Musculoskeletal Patients Using Smart Insole and Artificial Intelligence Gait Analysis. J. Cachexia Sarcopenia Muscle.

[35] Ko J.B., Kim K.B., Shin Y.S., Han H., Han S.K., Jung D.Y., Hong J.S. (2021). Predicting Sarcopenia of Female Elderly from Physical Activity Performance Measurement Using Machine Learning Classifiers. Clin. Interv. Aging.

[36] Chen H., Du N., Xiao H., Wang Z. (2025). Exploring the Link between the ZJU Index and Sarcopenia in Adults Aged 20-59 Using NHANES and Machine Learning. Sci. Rep..

[37] Luo X., Ding H., Broyles A., Warden S.J., Moorthi R.N., Imel E.A. (2023). Using Machine Learning to Detect Sarcopenia from Electronic Health Records. Digit. Health.

[38] Brockhattingen K.K., Karlsson E.H., Bielefeldt T.B.R., Naemi A., Andersen-Ranberg K., Moradbeiki P., Ebrahimi A., Wiil U.K. (2026). The Sarcopenia Artificial Intelligence Diagnostic Decision Support System (SAID DSS)—A Multimodal Deep Learning Model. BMC Geriatr..

[39] Balakrishnan M., Kumar J., Mathunny J.J., Karthik V., Devaraj A.K. (2025). SarcoNet: A Pilot Study on Integrating Clinical and Kinematic Features for Sarcopenia Classification. Diagnostics.

[40] Aznar-Gimeno R., Perez-Lasierra J.L., Perez-Lazaro P., Bosque-Lopez I., Azpiroz-Puente M., Salvo-Ibanez P., Morita-Hernandez M., Hernandez-Ruiz A.C., Gomez-Bernal A., Rodrigalvarez-Chamarro M.d.l.V. (2024). Gait-Based AI Models for Detecting Sarcopenia and Cognitive Decline Using Sensor Fusion. Diagnostics.

[41] Ozgur S., Altinok Y.A., Bozkurt D., Saraç Z.F., Akçiçek S.F. (2023). Performance Evaluation of Machine Learning Algorithms for Sarcopenia Diagnosis in Older Adults. Healthcare.

[42] Wu L.-W., OuYoung T., Chiu Y.-C., Hsieh H.-F., Hsiu H. (2022). Discrimination between Possible Sarcopenia and Metabolic Syndrome Using the Arterial Pulse Spectrum and Machine-Learning Analysis. Sci. Rep..

[43] Verma V.K., Verma S. (2022). Machine Learning Applications in Healthcare Sector: An Overview. Mater. Today Proc..

[44] Talwar A., Lopez-Olivo M.A., Huang Y., Ying L., Aparasu R.R. (2023). Performance of Advanced Machine Learning Algorithms over Logistic Regression in Predicting Hospital Readmissions: A Meta-Analysis. Explor. Res. Clin. Soc. Pharm..

[45] Pandey V., Verma P., Natesan G., Chaube U. (2025). Advancements in Breast Cancer Therapy: Integrating AI Tools for Drug Discovery and Clinical Trials. Comput. Biol. Med..

[46] Chou Y.-Y., Wang M.-S., Lin C.-F., Lee Y.-S., Lee P.-H., Huang S.-M., Wu C.-L., Lin S.-Y. (2024). The Application of Machine Learning for Identifying Frailty in Older Patients during Hospital Admission. BMC Med. Inform. Decis. Mak..

[47] Li W., Zeng L., Yuan S., Shang Y., Zhuang W., Chen Z., Lyu J. (2023). Machine Learning for the Prediction of Cognitive Impairment in Older Adults. Front. Neurosci..

[48] Çekok F.K., Alcan V. (2025). Predicting Risk of Falling in Older Adults Using Supervised Machine Learning: A Comparative Analysis of Model Performance. Z. Für Gerontol. Geriatr..

[49] Di Martino F., Delmastro F., Dolciotti C. (2023). Explainable AI for Malnutrition Risk Prediction from M-Health and Clinical Data. Smart Health.

[50] Jia Q., Pan Y., Pan D., Xia Y., Wu J. (2025). Application of Comprehensive Geriatric Assessment in Assessment, Management and Prognosis of Older Adult Patients with Sarcopenia: A Review of Research Progress and Practice. Gerontology.

[51] Lin F.-C., Chen P.-H., Yang C.-H., Yang C.-C., Chuang H.-Y., Hung C.-H. (2025). AI-Driven Fall Prediction across Generations: Integrating Deep Learning and Machine Learning for Young, Middle-Aged, and Older Adults. Gerontology.

[52] Leme D.E.d.C., de Oliveira C. (2023). Machine Learning Models to Predict Future Frailty in Community-Dwelling Middle-Aged and Older Adults: The ELSA Cohort Study. J. Gerontol. Ser. A.

[53] Melo B.R.d.S., Luchesi B.M., Barbosa G.C., Pott Junior H., Martins T.C.R., Gratão A.C.M. (2022). Agreement between Fragility Assessment Instruments for Older Adults Registered in Primary Health Care. Rev. Gauch. Enferm..

[54] Zhang C., Yan R., Liu X., Nie X., Peng Y., Peng X. (2026). Empirical Simulation of Internal Validation Methods for Prediction Models: Comparing k-Fold Cross-Validation with Bootstrap-Based Optimism Correction. J. Clin. Epidemiol..

[55] Jiang L., Xia Z., Zhu R., Gong H., Wang J., Li J., Wang L. (2023). Diabetes Risk Prediction Model Based on Community Follow-up Data Using Machine Learning. Prev. Med. Rep..

[56] Li L., Xia Z., Zeng X., Tang A., Wang L., Su Y. (2024). The Agreement of Different Techniques for Muscle Measurement in Diagnosing Sarcopenia: A Systematic Review and Meta-Analysis. Quant. Imaging Med. Surg..

[57] Nikodinovska V.V., Ivanoski S. (2023). Sarcopenia, More Than Just Muscle Atrophy: Imaging Methods for the Assessment of Muscle Quantity and Quality. RöFo-Fortschritte Auf Dem Geb. Röntgenstrahlen Bildgeb. Verfahr..

