# Supplementary material for: Exploring Artificial Intelligence Models for the Detection and Prediction of Sarcopenia: A Scoping Review
Source: J Clin Med. 2026 Oct 26;15(21):8320. doi: 10.3390/jcm15218320 (PMC13763701; doi:10.3390/jcm15218320)
Supplement: Supplementary file 1 [file jcm-15-08320-s001.zip › Supplementary Table S1.pdf]

Table S1. Search strategy

Database (February 28)	Total
PubMed<br>("Sarcopenia"[MeSH] OR sarcopenia[tiab] OR sarcopenic[tiab] OR sarcopaenia[tiab]) AND ("Artificial Intelligence"[MeSH] OR "Machine Learning"[MeSH] OR "Deep Learning"[MeSH] OR "artificial intelligence"[tiab] OR "machine learning"[tiab] OR "deep learning"[tiab] OR "neural network*"[tiab] OR "convolutional neural network*"[tiab])	537
Scopus<br>TITLE-ABS-KEY (sarcopenia OR sarcopenic OR sarcopaenia) AND TITLE-ABS-KEY ("artificial intelligence" OR "machine learning" OR "deep learning" OR "neural network*" OR "convolutional neural network*")	803
Web of Science<br>(TS=(sarcopenia OR sarcopenic OR sarcopaenia)) AND TS=("machine learning" OR "deep learning" OR "neural network*" OR "convolutional neural network*")	1.165
Scielo<br>((sarcopenia OR sarcopenic OR sarcopaenia)<br>AND<br>("machine learning" OR "deep learning" OR "neural network"))	1
LILIACS<br>(sarcopenia OR sarcopenic OR sarcopaenia) AND ("machine learning" OR "deep learning" OR "neural network" OR "convolutional neural network")	404
Total	2910
