# Supplementary material for: Exploring Artificial Intelligence Models for the Detection and Prediction of Sarcopenia: A Scoping Review
Source: J Clin Med. 2026 Oct 26;15(21):8320. doi: 10.3390/jcm15218320 (PMC13763701; doi:10.3390/jcm15218320)
Supplement: Supplementary file 1 [file jcm-15-08320-s001.zip › Supplementary Table S2.pdf]

Table S2: Diagnostic performance of machine learning models for sarcopenia detection, stratified by diagnostic consensus criterion

Diagnostic criterion	Studies	Median AUC (range)	Median sensitivity (range)	Median specificity (range)	Median F1-score (range)
AWGS2	21	0.871 (0.710–0.970) [n=18]	0.775 (0.441–1.000) [n=17]	0.882 (0.670–1.000) [n=16]	0.778 (0.392–1.000) [n=14]
EWGSOP2	6	0.924 (0.870–0.975) [n=4]	0.915 (0.800–1.000) [n=5]	0.922 (0.829–0.987) [n=4]	0.833 (0.748–0.921) [n=5]
FNIH	1	0.763 [n=1]	NR	NR	NR

Abbreviations: NR = not reported by the original study. AWGS2 = Asian Working Group for Sarcopenia, updated consensus; EWGSOP2 = European Working Group on Sarcopenia in Older People, revised criteria; FNIH = Foundation for the National Institutes of Health Sarcopenia Project.

Note: Analysis derived from the 28 studies included in the review. For each study, the first reported point estimate of AUC, sensitivity, specificity, and F1-score was extracted; for studies reporting a given metric stratified by subgroup (e.g., sex or temporal validation cohort), the first reported subgroup value was used, when both a validation and an independent test set were reported, the test set estimate was used. One study (Shin et al., 2025) reported the AUC separately for each diagnostic sub-criterion (SMI, handgrip strength, gait speed) without a combined overall AUC, and was therefore excluded from the AUC analysis only; its combined sensitivity, specificity, and F1-score (male model) were retained. These exclusions account for the variation in n across criteria and metrics. Values are summarized descriptively (median and range) within each diagnostic consensus criterion; no formal statistical test was performed given the small number of studies in some subgroups (FNIH, n=1) and the substantial heterogeneity in study design, population, and reporting completeness across the included literature.
