# Supplementary material for: Exploring Artificial Intelligence Models for the Detection and Prediction of Sarcopenia: A Scoping Review
Source: J Clin Med. 2026 Oct 26;15(21):8320. doi: 10.3390/jcm15218320 (PMC13763701; doi:10.3390/jcm15218320)
Supplement: Supplementary file 1 [file jcm-15-08320-s001.zip › Supplementary Table S3.pdf]

Table S3. TRIPOD+AI Reporting Compliance Across Included Studies on Machine Learning Models for Sarcopenia Diagnosis

	1	2	3a	3b	3c	4	5a	5b	6a	6b	6c	7	8a	8b	8c	9a	9b	9c	10	11	12a	12b	12c	12d	23b	24	25	26	27a	27b	27c
Ozgur et al., 2023																															
Yu et al., 2025																															
Luo et al., 2023																															
Li et al., 2024																															
Ko et al., 2021																															
Wang et al., 2024																															
Chong et al., 2025																															
Brockhattingen et al., 2026																															
Ryu et al., 2023																															
Chen et al., 2025																															
Wu et al., 2022																															
Wang et al., 2026																															
Huang et al., 2025																															
Liao et al., 2023																															
Aznar-Gimeno et al., 2024																															
Yang et al., 2025																															
Kim et al., 2023																															
Kim et al., 2024																															
Wang et al., 2025																															
Shin et al., 2025																															
Yang et al., 2025																															

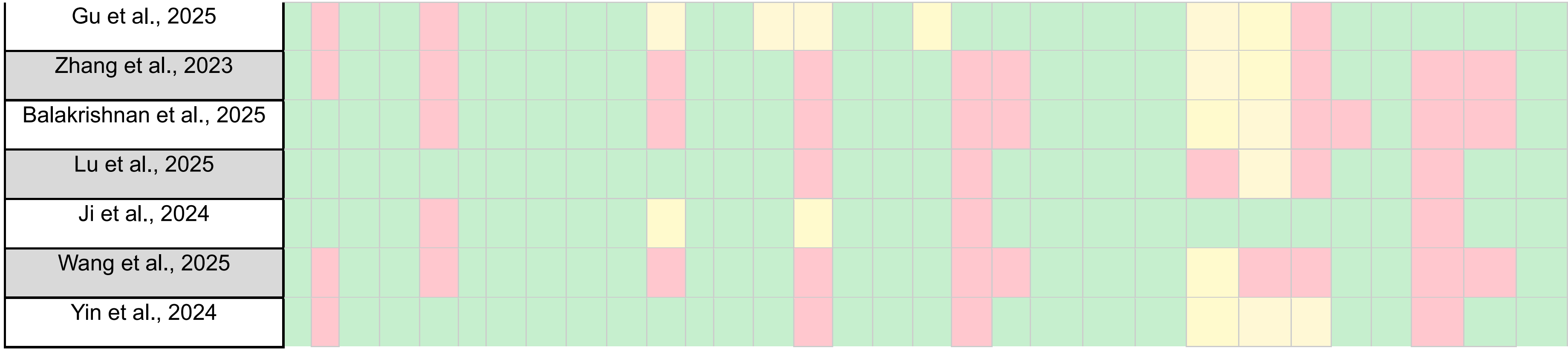

TRIPOD+AI checklist compliance across included studies. Each row represents an included study and each column corresponds to a numbered TRIPOD+AI item. Color coding indicates compliance status: green = item fulfilled, red = item not fulfilled, yellow = not applicable. Items are numbered according to the official TRIPOD+AI framework (Collins et al., BMJ 2024)
